# Whole-genome analysis of pseudorabies virus gene expression by real-time quantitative RT-PCR assay

**DOI:** 10.1186/1471-2164-10-491

**Published:** 2009-10-23

**Authors:** Dóra Tombácz, Judit S Tóth, Pál Petrovszki, Zsolt Boldogkői

**Affiliations:** 1 Department of Medical Biology, Faculty of Medicine, University of Szeged, Somogyi B. st. 4., Szeged, H-6720, Hungary

## Abstract

**Background:**

Pseudorabies virus (PRV), a neurotropic herpesvirus of pigs, serves as an excellent model system with which to investigate the herpesvirus life cycle both in cultured cells and *in vivo*. Real-time RT-PCR is a very sensitive, accurate and reproducible technique that can be used to detect very small amounts of RNA molecules, and it can therefore be applied for analysis of the expression of herpesvirus genes from the very early period of infection.

**Results:**

In this study, we have developed and applied a quantitative reverse transcriptase-based real-time PCR technique in order to profile transcription from the whole genome of PRV after lytic infection in porcine kidney cells. We calculated the relative expression ratios in a novel way, which allowed us to compare different PRV genes with respect to their expression dynamics, and to divide the PRV genes into distinct kinetic classes. This is the first publication on the whole-genome analysis of the gene expression of an alpha-herpesvirus by qRT2-PCR. We additionally established the kinetic properties of uncharacterized PRV genes and revised or confirmed data on PRV genes earlier examined by traditional methods such as Northern blot analysis. Our investigations revealed that genes with the same expression properties form clusters on the PRV genome: nested overlapping genes belong in the same kinetic class, while most convergent genes belong in different kinetic classes. Further, we detected inverse relationships as concerns the expressions of EP0 and IE180 mRNAs and their antisense partners.

**Conclusion:**

Most (if not all) PRV genes begin to be expressed from the onset of viral expression. No sharp boundary was found between the groups of early and late genes classified on the basis of their requirement for viral DNA synthesis. The expressions of the PRV genes were analyzed, categorized and compared by qRT2-PCR assay, with the average of the minimum cycle threshold used as a control for the calculation of a particular R value. In principle, this new calculation technique is applicable for the analysis of gene expression in all temporally changing genetic systems.

## Background

The pseudorabies virus (PRV; also called Aujeszky's disease virus or suid herpesvirus type 1) is a member of the alpha-herpesviruses and an important pathogen of pigs, causing Aujeszky's disease [1]. The PRV is a valuable model organism in herpesvirus research [2] and also a powerful tool in neuroscience, employed to map neural circuits [[3-5], and [6]] and gene delivery [7]. The PRV life cycle is primarily controlled at the level of transcription. The genes of α-herpesviruses are divided into three major temporal classes (immediate-early, IE or α; early, E or *β*, and late, L or γ), which are regulated in a coordinated, cascade-like fashion [[8] and [9]]. First, the IE genes are expressed, independently of *de novo *protein synthesis from the virus. The products of these genes are transcription factors and other regulatory proteins. PRV IE180 protein [homologous to herpes simplex virus (HSV) ICP4 protein] is the major regulator of the transcription of the E genes, which in turn are mainly involved in replication of the viral DNA. Blockers of DNA synthesis inhibit the E gene expression to a much lesser extent than the L gene expression. On a finer scale, the E genes can be subdivided into *β*1 (E) and *β*2 (E/L) genes. Finally, the L genes are transcribed, encoding mainly structural proteins involved in virion assembly. The expression of the L genes is partially (γ1, leaky L genes) or completely (γ2, true L genes) dependent on the viral DNA replication. HSV encodes 5 IE genes: *icp0*, *icp4*, *icp22*, *icp27 *and *icp47*. By contrast, PRV has been reported to express *ep0 *(homologous to *icp0 *of HSV; [10] and *ul54 *(the homolog of *icp27 *of HSV; [11]) proteins in the E kinetics, and it lacks the *icp47 *gene. The *ie180 *gene of PRV has been shown to be a true IE gene in various experimental systems [[12] and [13]]; however, there is no consensus as to whether the US1 protein (Rsp40; homologous to HSV ICP22) is expressed in the IE [14] or the E kinetics [15]. Herpesviruses are capable of two types of infections: lytic (or productive) and latent [2]. In the lytic pathway, the entire transcription machinery of the herpesvirus is initiated, and the progress of infection eventually leads to the production of new virions and the lysis of infected cells. In contrast, in latency, only a limited segment (LAT region) of the herpesvirus genome is transcriptionally active, no new virus particles are produced and the cells survive the infection. A characteristic feature of the organization of the herpesvirus genome is the presence of nested genes producing 3'-coterminal transcripts. The read-through of overlapping genes is regulated by the ICP27 protein, which helps in the recognition of the internal polyA signals, resulting in differential transcript lengths [16]. The gene expression of α-herpesviruses, such as HSV types 1 and 2, Varicella-zoster virus, bovine herpesvirus type 1 and PRV has been investigated earlier by traditional methods including Northern blot analysis, ribonuclease protection assay, and end-point RT-PCR analysis. However, each of these techniques is associated with a number of disadvantages. For example, Northern blot analysis is labor-intensive, allows only semiquantitative determination of the mRNA level and is unsuitable for multiple mRNA analysis. Moreover, hybridization-based membrane arrays profile changes in a nonlinear fashion, tending to overemphasize large alterations, and they are insensitive to smaller variations. Ribonuclease protection assay-based methods require the use of polyacrylamide gel electrophoresis and typically utilize radioactively labeled probes. The limitations of endpoint PCR technique are the time-consuming procedure, the poor precision and the variable endpoints between samples. Over the past few years, microarray techniques have revolutionized practically all disciplines of molecular biology, including herpesvirus research. As compared with traditional methods, microarray analysis is superior in that it is applicable for the simultaneous analysis of a large number of genes, and even whole genomes. The disadvantage of DNA chip technology is associated with the uncertain quality control: it is impossible to assess the identity of DNA immobilized on any microarray. Further, there are many artifacts associated with image and data analysis. Real-time RT-PCR is an alternative to microarray techniques for the analysis of transcription from multiple genes. The main advantage of real-time PCR is that it is more sensitive to low-fold changes than other high-throughput assays. In addition, the real-time PCR technique provides a reproducible quantitation of DNA copies, and has a large dynamic range, and various controls can be included to ensure accuracy, such as a loading control to verify equal cDNA loading, a no-primer control to prove a measure of non-amplification-related background, a no-template control to screen for contamination of reagents or false amplification, and a no-RT control with confirm the absence of DNA contamination. In contrast to microarray techniques, in real-time PCR the parameters for each gene can be optimized individually. Moreover, the identity of PCR products can be confirmed through melting curve analysis, restriction endonuclease analysis, or DNA sequencing. In fact, real-time PCR is often used to verify gene expression data obtained by microarrays. Nevertheless, considerable pitfalls may be associated with this technique. The major limitations of real-time RT-PCR relative to microarray techniques are the higher cost and labor intensiveness for a large number of samples. Another disadvantage of real-time PCR as compared with blotting techniques is that only the accumulation, but not the size of the transcripts can be monitored. Microarray techniques have recently been applied to investigate herpesvirus gene expression [[17-19] and [20]]; to analyze the effects of the deletion of particular viral genes or of the specific experimental conditions on whole-genome viral gene expression [[21,22], and [23]]; and to analyze the impact of virus infection on the expression of cellular genes [[24-26] and [27]]. The expression of PRV genes has been studied by traditional methods, but many its genes have not yet been characterized at all. Flori and co-workers [27] investigated the dialog between PRV and epithelial cells, but obtained poor resolution for viral transcripts that did not provide conclusive data on the temporal expression of the PRV genes. To date, Real-time RT-PCR has not been frequently utilized in herpesvirus research for global gene expression analysis. With this technique Oster and Höllsberg [28] carried out a kinetic analysis of 35 genes of human herpesvirus 6B, a *β*-herpesvirus, and Dittmer and colleagues [29] performed a whole-genome profiling of the rhesus monkey rhadinovirus, a γ-herpesvirus. As far as we are aware, no genome-wide expression data obtained by qRT2-PCR have been published so far on α-herpesviruses. In the present study, we describe the development and utilization of a real-time RT-PCR assay for the global analysis of PRV gene expression. We applied a novel method that allows the kinetic characterization of individual PRV genes, and also the comparison of the expression dynamics of different viral genes. Expression profiles were constructed on the basis of relative expression ratios (Rs) calculated as described in the Materials section. This method is applicable for the analysis of gene expression in any genetic system that progressively changes in time.

## Results

### Experimental design

**Figure 1:**
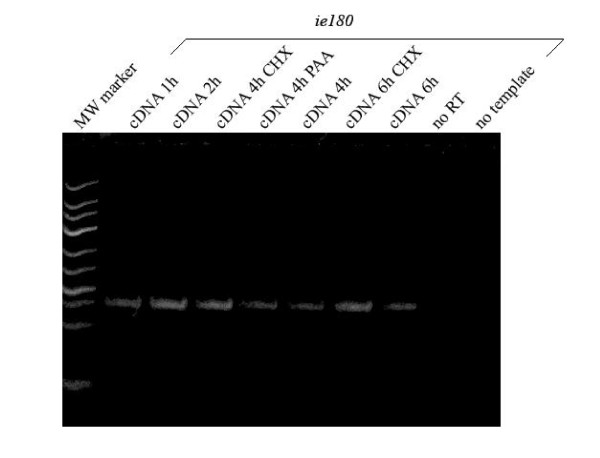
**Controls for qRT^2^-PCR**. Polyacrylamide gel electrophoresis of the products from 30 cycles of real-time PCR on cDNA derived from PK-15 cells infected by the PRV for 1, 2, 4 or 6 h or from cells infected by the virus for 4 or 6 h and treated with CHX or PAA. A no-template control was used for each primer pair, consisting of water, to test for false-positive results, and no-RT control was used to ensure the absence of DNA contamination. The GeneRuler™ Low Range DNA Ladder (Fermentas) is shown at the left side of the photo. The cDNAs of *ie180 *gene (a) and *ul30 *gene (b) were used for the presentation.

For each gene, a minimum of 3 independent replicates were carried out for statistical confidence, and the median of these values along with the standard error was calculated. In these experiments, porcine kidney (PK)-15 epithelial cells were infected with PRV with a low multiplicity of infection [MOI; 0.1 plaque-forming units (pfu)/cell]. The reason for this was that our preliminary experiments indicated that infecting cells with a low dose of the virus produced a much better resolution of the cycle threshold (Ct) values for PRV genes at different time points than infecting with a higher MOI (data not shown). However, the use of a low MOI led to a larger proportion of the cells remaining uninfected; accordingly, in order to avoid the initiation of a new infection cycle we chose a relatively short maximal incubation period. Initially, we attempted an 8-h investigation period, but observed a second infection wave of the virus (data not shown), and therefore we restricted our analysis to 6 h post-infection (pi). The transcription of PRV genes was monitored at 5 time points: 0, 1, 2, 4 and 6 h. Prior to PRV infection, cells were either untreated or treated with cycloheximide (CHX), a protein synthesis blocker, or phosphonoacetic acid (PAA), an inhibitor of DNA synthesis. We used strand-specific primers for the reverse transcription, one of the reasons for this being that we intended to exclude the distorting effects of potential overlapping antisense RNAs transcribed from the antiparallel DNA strands, which cannot be distinguished by oligo-dT- or random priming-based RT (this issue will be described elsewhere). Another reason was that it produced a much higher amount of specific cDNAs than oligo-dT priming (data not shown), which was especially important under our experimental conditions with a low MOI. We utilized the mathematical model for relative quantification described by Soong et al. [30]; however, we calculated the R values in a different way: we used the average of the maximal E^Ct-sample ^values for each gene as a control, which was normalized with the average of the corresponding 28S values (E^Ct-reference^). Thus, in our calculation, a particular R value indicates the ratio of the mRNA level of a gene at a given time point to the maximal RNA level of this gene (maximal gene expression occurred at 6 h pi for all genes except *us3 *and *llt2*, which peaked at 4 h and at 2 h pi, respectively). The relative amounts of the transcripts of different genes cannot be compared because the primer efficiencies may vary both in RT and in PCR. Even so, use of the maximal expression as the control value allows a comparison of the expression dynamics of viral genes both at individual time points and across the entire infection period. The specificity of the PCR products was confirmed by quality control experiments, including Tm analysis and PAGE (formation of a single product with the appropriate molecular weight was accepted); if some doubts remained, the amplification products were subjected to DNA sequencing or restriction endonuclease analysis using amplicons containing recognition sites for a particular restriction endonuclease [see Additional file 1]. The accuracy of sampling and qRT2-PCR analysis were ensured by using loading controls, as well as no-RT, no-primer and no-template controls (Figure 1).

### Reproducibility of the internal control

**Figure 2:**
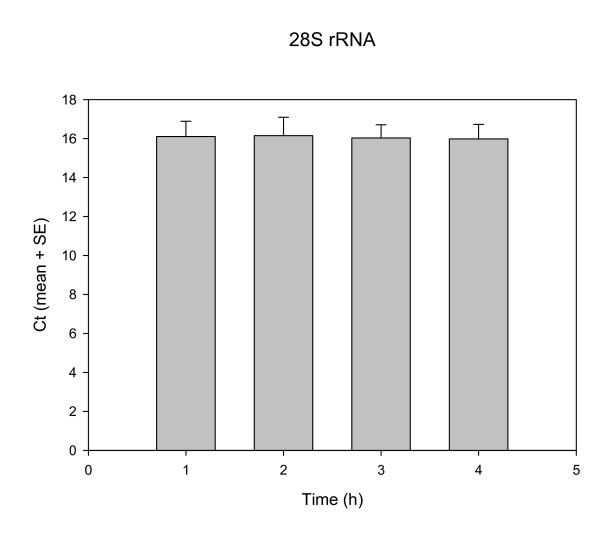
**Reproducibility of the reference control**. The constancy of the 28S rRNA level (shown by the similar Ct values) throughout the 6-h infection period indicates that this gene serves as an appropriate reference control.

A major concern regarding the choice of internal reference gene control in viral gene expression studies is the constancy of the reference gene products throughout the entire examination period. We tested whether the 28S ribosomal RNA of the pig fulfilled this requirement, and found that this gene was suitable as reference gene for the study of PRV infection, at least in our 6-h examination period. The 28S rRNA values were highly reproducible: the mean value ± SD of all measurements the experiments was 8.03 ± 0.61 cycles throughout (Figure 2).

### Classification of PRV genes in terms of dependence on de novo protein synthesis and DNA replication

Traditionally, lytic herpesvirus genes are classified into distinct kinetic groups on the basis of the effects of protein and DNA synthesis inhibition on the gene expression. In general, inhibitory drugs are applied to infected cells for a prolonged incubation period (24, 48 or 72 h; [[31,32] and [33]]. Instead, similarly to Stingley *et al *[18], we used shorter incubation periods (2, 4 and 6 h for CHX, and 4 and 6 h for PAA) and compared the inhibitory effects of these drugs by calculating the R value ratios for the treated and untreated samples.

#### CHX analysis

**Figure 3:**
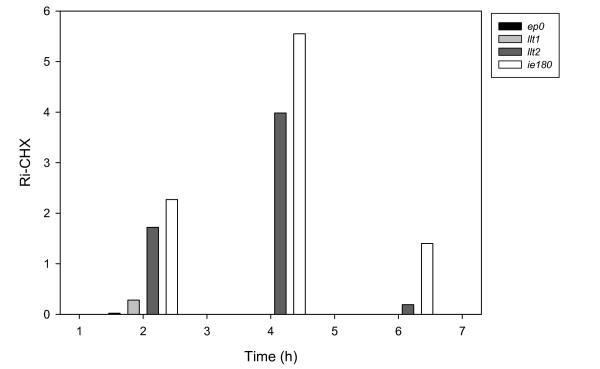
**Effects of the CHX on the expression of *ie180*, *llt1*, *llt2 *and *ep0 *genes**. This Figure shows the dependence of the PRV gene expression on *de novo *protein synthesis on two genes (*ie180 *and *ep0*) and two antisense transcripts (*llt1 *and *llt2*) overlapping these genes. PK-15 cells infected with PRV were incubated in the presence or absence of CHX for 2, 4 or 6 h. The CHX-mediated inhibition of viral gene expression (R_i-CHX_) was calculated as follows: R_i-CHX _= R_CHX_/R_UT_, where R_CHX_is the R value of the CHX-treated samples; and R_UT_is the R value of the untreated samples.

To test the requirement of *de novo *protein synthesis for PRV mRNA production, PK-15 cells were untreated or treated with 100 *μ*g/ml CHX prior to the infection of cells with the virus. At the indicated time points, RNA was isolated and converted to cDNA, which was subsequently analyzed by qRT2-PCR. The degree of inhibition [1-R_i-CHX_) × 100] was found to range between 97.3 and 100% for all but one protein encoding PRV gene (the ie180 gene) and two antisense transcripts [the long-latency transcript-1 (llt1), antiparallel to ep0, and the long-latency transcript-2 (llt2), antiparallel to ie180). IE180 mRNA displayed a significantly increased level of expression in the CHX-treated samples at the analyzed 3 time points: 2.27-fold at 2 h; 5.55-fold at 4 h; 1.4-fold at 6 h pi (Figure 3). We explain this phenomenon in that the IE180 proteins exert an inhibitory effect on their own synthesis (upon the binding of their own promoters; [34]), which is resolved by CHX blocking protein synthesis from the IE180 mRNAs. The other exception for the negative CHX effect is *llt2*: a 1.72-fold increase at 2 h and a 3.983-fold increase at 4 h. Interestingly, at 6 h pi *llt2 *is significantly repressed by CHX. The repression of *llt1 *expression at 2 h pi is relatively low (R_i-CHX-2h _= 0.281), but this antisense transcript is significantly blocked by CHX at 4 and 6 h pi. Overall, our CHX analysis indicated that the only true IE protein-encoding gene of PRV is *ie180*. While the *icp27 *and *icp0 *genes in the HSV are IE genes, their PRV homologs *ul54 *[11] and *ep0 *[10] genes were earlier described as E genes, which was confirmed by our CHX analysis. The *icp22 *gene of HSV is expressed with IE kinetics; our analysis revealed that its homologous counterpart, the *us1 *gene of PRV, is significantly blocked by CHX, and hence it is not an IE gene. As a result of CHX treatment, LLT2 exhibits elevated levels at 2 and 4 h pi, which indicates that IE180 transcription factor exerts a negative effect on its expression. Thus, LLT2 appears to be an IE transcript. However, *llt2 *displays fairly low relative expression at 1 h pi [see Additional file 2], and therefore it cannot be regarded as an IE gene beyond doubt.

#### PAA analysis

**Figure 4:**
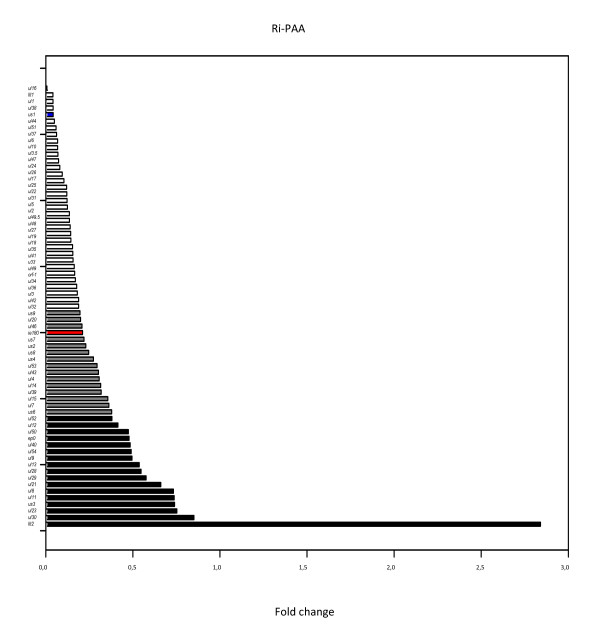
**Clustering of genes on the basis of the effects of PAA on gene expression**. Horizontal bars show the PRV genes ranked on the basis of R_i-PAA _values in an increasing sequence. Early and late gene clusters were separated by a set of genes labeled as E/L using arbitrarily given threshold values.

For examination of the dependence of the PRV genes on DNA replication, PK-15 cells were infected with the virus in the absence or presence of 400 *μ*g/ml PAA, an inhibitor of DNA polymerase. It was expected that PAA would exert a more drastic effect on the L genes because the expressions of these genes are highly dependent on DNA replication. However, PAA must affect the E gene expression, too. The reason for this is that the gene expression is dependent not only on the promoter activity, but also on the copy numbers of the genes, which are higher after DNA replication than in the initial phase of infection when the PRV DNA is represented in a single copy in a cell (at least in our system). The inhibitory effect of PAA on gene expression was calculated by using the ratio of the R values for the PAA-treated/untreated samples at 6 h pi (R_i-PAA _= R_6h-PAA_/R_6h-UT_): a low ratio indicates a strong inhibitory effect and vice versa. The PRV genes were ranked from the least inhibited (highest R_i-PAA_) to the most inhibited (lowest R_i-PAA_). We expected that such a ranking of the PRV genes on the basis of the inhibitory effect would provide natural clusters of E and L genes. Indeed, the results obtained conformed fairly well to the data published earlier on HSV and PRV genes, though there is a certain degree of disagreement between the data published by the different authors on the HSV genes, and only incomplete expression data are available for the PRV genes. Moreover, in many cases a PRV gene is characterized without comparative analysis, e.g. "it appears 3 h pi". For comparison of the HSV and PRV genes, we used five main sources for the HSV data: microarray data published by Wagner [35,36]; various expression data compiled by Roizman [37,38]; and the data collected by Mettenleiter [39]. For the PRV genes, we used the data compiled by Mettenleiter [39]. We classified the analyzed but uncategorized PRV genes via the following principles: if the mRNA was detected between 1 and 2 h pi,, between 3 and 4 h pi, or 5 h pi, the particular gene was classified as E, E/L or L, respectively. In the upper part of the ranking list (Table 1, Figure 4), [see Additional file 3], we mostly find typical E genes, with typical L genes in the bottom part of the list. However, we could not draw a clear-cut line between the E and L genes; instead, we put the 15 genes with intermediate values in the E/L group. The precise locations of borderlines between kinetic classes were drawn arbitrarily, because we found smooth transitions between the R_i-PAA _values. We chose a typical E gene (ul52) of the HSV as the last member of the E group, and a typical L gene (ul32) of the HSV as the first member of the L group. The 3 protein-encoding genes with the highest R_i-PAA _values were ul30 (0.851), ul23 (0.752) and us3 (0.739). These high R_i-PAA _values indicate that PAA does not exert a significant cytotoxic effect on the gene expression apart from blocking DNA replication. The 2 LLT transcripts display a surprising response to PAA treatment: the level of LLT1 increases to 2.94-fold at 4 h, and drops to 0.007-fold at 6 h pi relative to the untreated sample; while the level of LLT2 increases markedly close to 40-fold at 4 h, and 3-fold at 6 h pi (this phenomenon will be discussed later). The genes with the lowest R_i-PAA _values are all but one (us1: 0.042) L genes in the HSV: ul16 (0.000), ul1 (0.041) and ul38 (0.042). The strong inhibitory effect of PAA on the us1 expression is surprising because it is an IE gene in the HSV. The ie180 gene expression is also significantly inhibited by PAA (0.211); however, we found that ie180 and to a certain extent us1 were expressed in an "irregular" manner in other analyses, too (see later). ul11 and ul21, which are L genes in HSV, do not appear to belong in the E kinetic class. Both genes were characterized as L genes in the PRV by De Wind et al. [59] using Northern blot analysis. Furthermore, ul13 is an L gene in the HSV, but its transcripts were detected at 2 h pi in the PRV. The ul2 and ul5 genes are E genes in the HSV, but were characterized as E/l and L genes, respectively, in the PRV by Dean and Cheung [55,60], respectively, which is in agreement with our results. We classified the kinetically still uncharacterized PRV genes as follows; E genes: us3, ul29 and ul30, L genes: ul17, ul18, ul27, ul32, ul33, ul35, ul37 and ul41. Peaking of the us3 gene expression at 4 h can be explained by the important role of US3 protein kinase in blocking the apoptotic pathway of the compromised cell in the E phase of infection [61]. Our kinetic classifications accorded more strongly with those published on the HSV than with those on the PRV. The reason may be that the expression profiling of PRV genes was performed by others with low resolution techniques. Our data allow the following conclusions: (1) there is no sharp boundary between the E and L genes; (2) classification of the PRV genes on the basis of the R_i-PAA _values yielded similar results those for the homologous HSV genes. A noteworthy exception is the us1 gene, which according to our PAA analysis is expressed in L kinetics; (3) the ie180 and llt genes cannot be classified on the basis of the R_i-PAA _data alone, because they display unique expression kinetics; (4) LLT1 and LLT2 respond differently to CHX and PAA treatment, indicating that these antisense transcripts are, at least partially, under different regulation. We assume that the putative antisense promoter (ASP; [10]) controls the expression of LLT2.

**Table 1:** Function and kinetic grouping of PRV genes

**Name**			**Transcription Kinetics**					**Function(s)^b^**	**References**
	**Wagner^37^**	**Wagner^38^**	**Roizman^39^**	**Roizman^40^**	**Mettenleiter^41^**	**Mettenleiter^41^**	**Our data^a^**		
*orf-1*	-	-	-	-	-	ND	L	unknown	
ul54 (*ICP27)	IE	IE	IE	IE	IE	E	E	*transcription regulation*	[40]
*ul53 *(gK)	L	EL	L	L	L	(EL) 3 h pi	E/L	viral egress	[40]
*ul52*	E	E	E	E	E	(E) 2 h pi	E	*DNA replication*	[40]
*ul51*	L	EL	L	L	L	E	L	viral egress/oralgen unknown	[40]
ul50 (*dUTPase)	E	E	E	E	E	(EL) 3 h pi	E	dUTPase, viral replication	[27]
*ul49.5 *(gN)	L	L	L2	L2	L	L	L	virion entry	[39]
ul49 (*VP22)	E?	E	L	L	E	(EL) 4 h pi	L	virion formation, tegumentation	[27]
ul48 (*VP16, IE-TIF)	?	EL	L	L	L	(L) 8 h pi	L	gene regulation, viral egress	[41]
ul47 (*VP13/14)	E	EL	L2	L2	L	L	L	secondary envelopment	[41]
ul46 (*VP11/12)	E	EL	L	L	L	E	E/L	unknown function, tegument protein	[41]
*ul27 *(gB)	E	EL	L1	L1	E	ND	L	cell-cell spread, virus entry	[27]
ul28 (*ICP18.5)	E	EL	L	L	E	E	E	DNA cleavage and packaging	[27]
ul29 (*ICP8)	E	E	E	E	ND	ND	E	DNA replication	[27]
*ul30*	E	E	E	L	E	ND	E	*DNA replication*	[27]
*ul31*	L	EL	L2	L2	L	(L) 6 h pi	L	nuclear egress	[42]
*ul32*	L	EL	L2	L2	L	ND	L	*DNA packaging*	[2]
*ul33*	L	EL	?	L	L	ND	L	*encapsidation of viral DNA*	[43]
*ul34*	L	L	?	L	ND	(EL) 3 h pi	L	nuclear egress	[14]
ul35 (*VP26)	L	L	L2	L2	L	ND	L	*capsid protein*	[44]
ul36 (*VP1/2)	?	EL	L2	L2	L	(EL) 3-9 h pi	L	tegumentation and egress	[45]
*ul37*	L	E	L	L	L	(E) 2 h pi	L	secondary envelopement, egress	[45]
ul38 (*VP19c)	L	L	L2	L2	L	(E) 2 h pi	L	*capsid protein*	[46]
*ul39 *(RR1)	E	E	E	E	E	(E) E/L	E/L	nucleotide synthesis	[46]
*ul40 *(RR2)	E	E	E	E	E	E	E	nucleotide synthesis	[47]
*ul41 *(VHS)	L	EL	L	L	L	ND	L	*RNase, gene regulation*	[48]
*ul42*	L	E	E	E	E	L	L	DNA replication	[47]
*ul43*	L	EL	?	L	E	E	E/L	unknown	[49]
*ul44 *(gC)	L	L	L2	L2	L	L	L	viral entry, virion attachment	[50]
*ul26*	E	L	L	L	L	L	L	scaffold protease	[52]
*ul25*	L	EL	L	L	L	L	L	capsid protein	[51]
ul24 (*VP24)	L	L	L	L	L	L	L	*unknown*	[52]
*ul23 *(TK)	E	E	E	E	E	E	E	nucleotide synthesis	[47]
*ul22 *(gH)	L	L	L2	L2	L	L	L	viral entry, cell-cell spread	[47]
*ul21*	L	E	?	L	L	L	E	capsid maturation	[47]
*ul20*	L	EL	L	L	L	ND	E/L	capsid transport	[2]
ul19 (*VP5)	L	EL	L1	L1	L	(L) 16 h pi	L	*capsid protein*	[53]
ul18 (*VP23)	L	EL	L	L	L	ND	L	*capsid protein*	[27]
*ul17*	L	EL	L	L	L	ND	L	*DNA cleavage and encapsidation*	[2]
*ul16*	?	EL	?	L	L	ND	L	*unknown, interacts with UL11*	[27]
*ul15*	L	EL	L	L	L	ND	E/L	*DNA cleavage and encapsidation*	[27]
*ul14*	L	EL	?	L	L	L	E/L	*DNA cleavage and packaging*	[47]
ul13 (*VP18.8)	L	EL	L	L	L	(E) EL	E	protein phosphorylation	[47]
ul12 (*AN)	E	E	E	E	E	(E) EL	E	alkaline nuclease	[47]
*ul11*	L	EL	L(?)	L	L	L	E	secondary envelopement	[47]
*ul10 *(gM)	L	E	L	L	L	E	L	egress, secondary envelopement	[54]
ul9 (*OBP)	E	E	L(?)	E	E	E	E	*ori depentent DNA synthesis*	[54]
ul8 (*OBPC)	E	E	E	E	E	(E/L) 3-5 h pi	E	*DNA replication*	[54]
*ul7*	?E	E	?	ND	L	(E/L) dE	E/L	unknown	[54]
*ul6*	L	E	?	ND	L	(E/L) dE	L	*capsid protein, portal protein*	[54]
*ul5*	E	E	E	E	E	(L) 6 h pi	L	*DNA replication*	[55]
*ul4*	E	E	?	L	L	(L) 6 h pi	E/L	*unknown*	[55]
*ul3.5*	-	-	-	-	-	E/L	L	replication, cell-to-cell spread	[55]
*ul3*	L	L	L2	L2	L	E/L	L	unknown	[55]
ul2 (*UNG)	E	E	E	L	E	E/L	L	*DNA repair*	[55]
*ul1 *(gL)	E	EL1	L	L	L	E/L	L	viral entry, cell-to-cell spread	[55]
ep0 (*ICP0)	IE	IE	IE	IE	E	E	E	gene regulation	[10]
*llt1*	L	ND	ND	ND	ND	ND	L	latency	[56]
*llt2*	L	ND	ND	ND	ND	ND	E	latency	[56]
ie180 (*ICP4)	IE	IE	IE	IE	IE	IE	E/L	gene regulation	[13]
us1 (*RSp40/ICP22)	IE	IE	IE	IE	IE	IE	L	*regulator of gene expression*	[27]
*us3*(PK)	E	E	E	E/L	E	E	E	nuclear egress	[27]
*us4*(gG)	E	E	L	L	E/L	E	E/L	unknown	[57]
*us6 *(gD)	L	E	L1	E/L	E/L	ND	E/L	entry	[27]
*us7 *(gI)	E	E	L	L	L	(L) 6 h pi	E/L	cell-to-cell spread	[27]
*us8 *(gE)	E	E	L2	L2	L	E	E/L	cell-to-cell spread	[58]
*us9 *(*11*K*)	E	E	?	L	L	L	E/L	anterograde spreed of virus	[58]
*us2 *(*28*K*)	E	E	?	L	L	(L) 5 h pi	E/L	*unknown*	[27]

This Table demonstrates the function and kinetic classification of PRV genes in comparison with available data on HSV and PRV genes.

^a ^PRV genes were classified on the bases of the ratio of the PAA-treated and untreated samples, at 6 h pi.

^b ^Gene functions in italics rely on studies of the HSV-1 homologs.

### Kinetics of PRV gene expression in untreated cells

Real-time RT-PCR is a high-resolution technique, which allows a detailed analysis of gene expression dynamics and a reliable temporal classification of viral mRNAs without drug treatment. The data from these experiments are presented in detail in the text [see Additional file 2 and 4]. The *us3 *PRV gene peaks at 4 h, *llt2 *at 2 h, while the other genes reach their maximum at 6 h within the 0-6 h examination period.

#### 0-6 h incubation period

**Figure 5:**
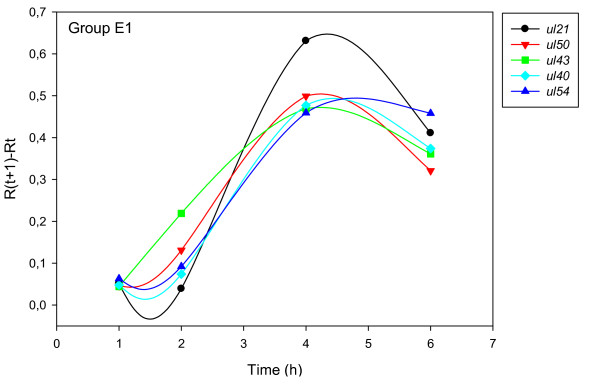
**Grouping and plotting of PRV genes using their R_Δ _values**. Pearson's correlation analysis was performed to group the PRV genes into clusters on the basis of similar expression dynamics (using pairwise R_Δ _values) over the four time intervals.

**Figure 6:**
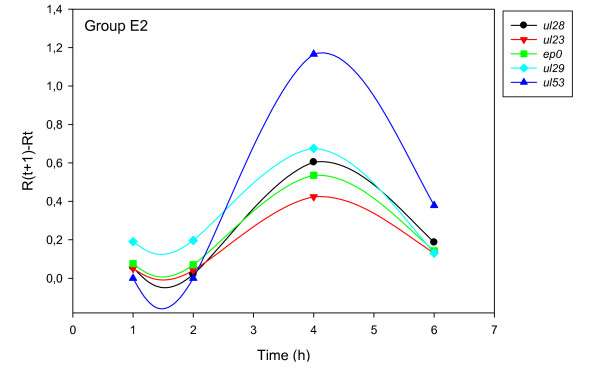
**Grouping and plotting of PRV genes using their R_Δ _values**. Pearson's correlation analysis was performed to group the PRV genes into clusters on the basis of similar expression dynamics (using pairwise R_Δ _values) over the four time intervals.

**Figure 7:**
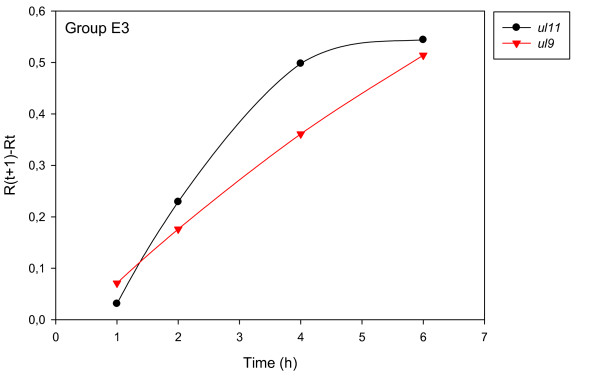
**Grouping and plotting of PRV genes using their R_Δ _values**. Pearson's correlation analysis was performed to group the PRV genes into clusters on the basis of similar expression dynamics (using pairwise R_Δ _values) over the four time intervals.

**Figure 8:**
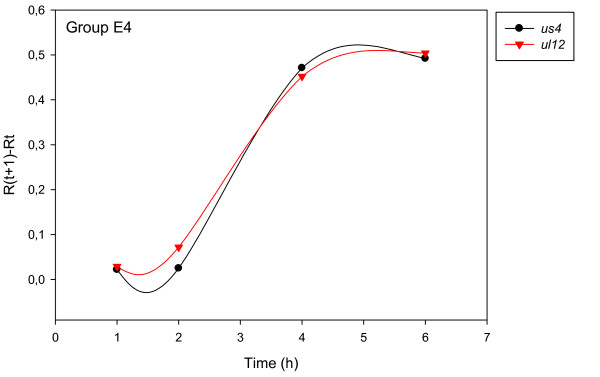
**Grouping and plotting of PRV genes using their R_Δ _values**. Pearson's correlation analysis was performed to group the PRV genes into clusters on the basis of similar expression dynamics (using pairwise R_Δ _values) over the four time intervals.

**Figure 9:**
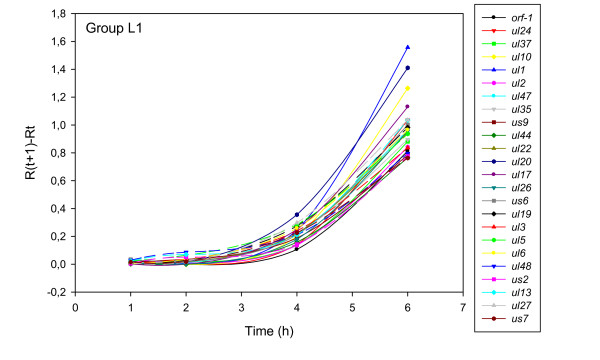
**Grouping and plotting of PRV genes using their R_Δ _values**. Pearson's correlation analysis was performed to group the PRV genes into clusters on the basis of similar expression dynamics (using pairwise R_Δ _values) over the four time intervals.

**Figure 10:**
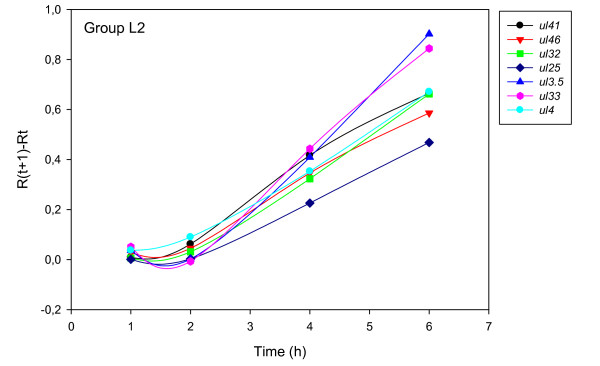
**Grouping and plotting of PRV genes using their R_Δ _values**. Pearson's correlation analysis was performed to group the PRV genes into clusters on the basis of similar expression dynamics (using pairwise R_Δ _values) over the four time intervals.

**Figure 11:**
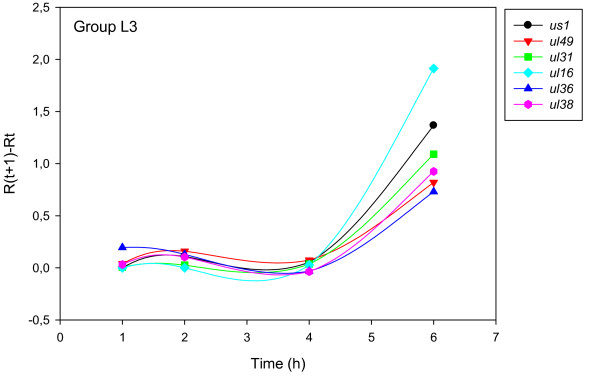
**Grouping and plotting of PRV genes using their R_Δ _values**. Pearson's correlation analysis was performed to group the PRV genes into clusters on the basis of similar expression dynamics (using pairwise R_Δ _values) over the four time intervals.

**Figure 12:**
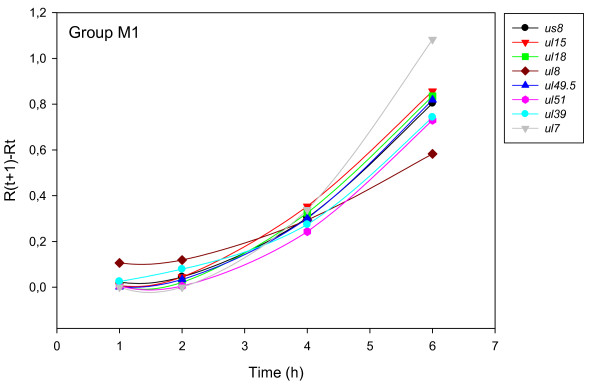
**Grouping and plotting of PRV genes using their R_Δ _values**. Pearson's correlation analysis was performed to group the PRV genes into clusters on the basis of similar expression dynamics (using pairwise R_Δ _values) over the four time intervals.

**Figure 13:**
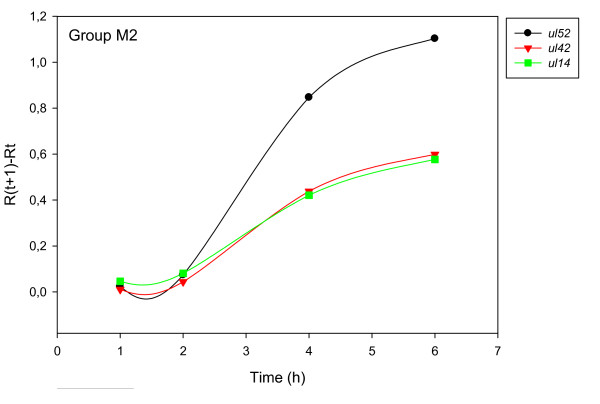
**Grouping and plotting of PRV genes using their R_Δ _values**. Pearson's correlation analysis was performed to group the PRV genes into clusters on the basis of similar expression dynamics (using pairwise R_Δ _values) over the four time intervals.

**Figure 14:**
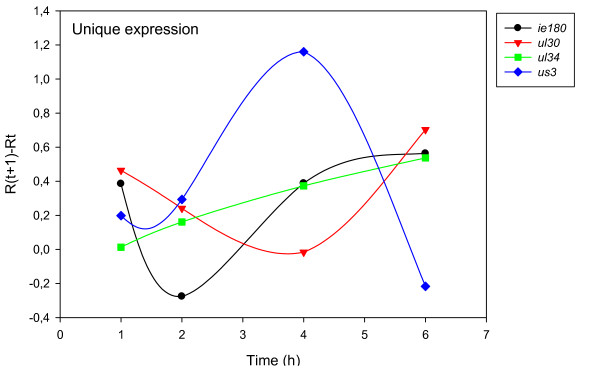
**Grouping and plotting of PRV genes using their R_Δ _values**. Pearson's correlation analysis was performed to group the PRV genes into clusters on the basis of similar expression dynamics (using pairwise R_Δ _values) over the four time intervals.

**Figure 15:**
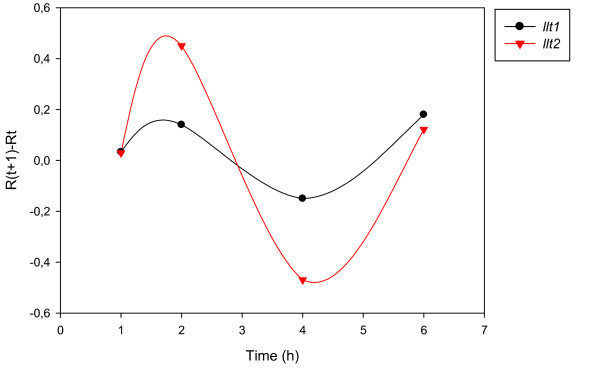
**Grouping and plotting of PRV genes using their R_Δ _values**. Pearson's correlation analysis was performed to group the PRV genes into clusters on the basis of similar expression dynamics (using pairwise R_Δ _values) over the four time intervals.

**Figure 16:**
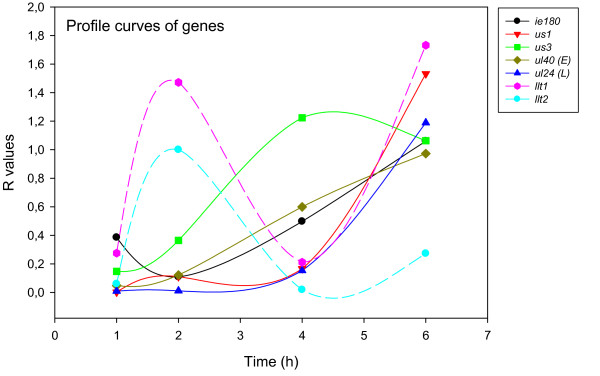
**Expression kinetics of some selected genes via their R values**. This Figure shows the expression curves involving the R values of the only IE (*ie180*) gene, a typical E gene (*ul40*) and a typical L (*ul24*) gene, two genes with irregular expression profiles: *us1 *(local minimum at 4 h pi) and us3 (maximal expression at 4 h pi), and *llt1 *and *llt2*.

**Figure 17:**
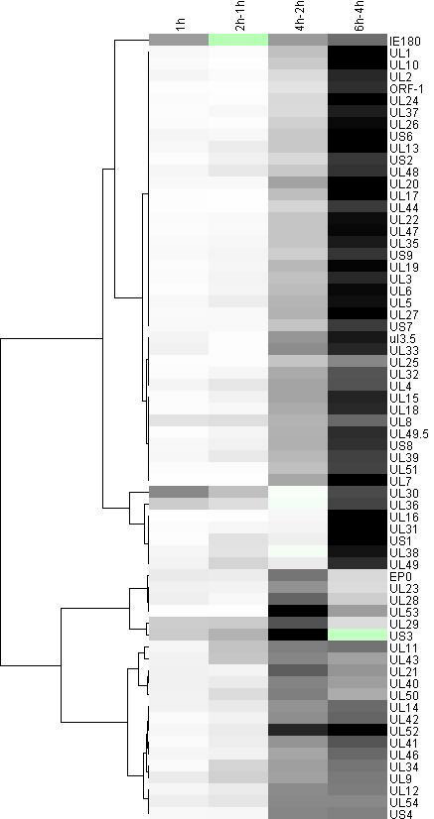
**Clusters of PRV R_Δ _values identified by the hierarchical clustering method**. The dendogram depicts the whole-genome profiling of protein-coding PRV genes, following *de novo *infection of PK-15 cells. Green indicates negative R_Δ _values, white low R_Δ _values, gray intermediate R_Δ _values, and black the highest level of increase in viral mRNA detected between two time points.

The accurate and reproducible data generated by real-time PCR allow the analysis of gene expression throughout the entire time course of infection. In order to classify genes on the basis of their expression kinetics, we performed Pearson's correlation analysis for each gene pair of the PRV, using the net increase (R_Δ_) in each time interval for the comparison [see Additional file 4b]. Genes were clustered into the same group if their pairwise correlation coefficients were high, ranging from r ≥ 0.9 to 0.99 depending on the group. Surprisingly, genes belonging in a particular group very rarely give similarly high values as any of the genes belonging in other groups. Albeit Pearson's coefficient expresses the kinetic properties of a gene in single numbers, the expression curves of genes belonging in the same group proved to be very similar (Figures 5, 6, 7, 8, 9, 10, 11, 12, 13, 14, 15). We distinguished 10 gene sets. Group L1 of PRV genes (each pair shared r ≥ 0.989 values) contains 24 genes, of which 22 were identified as L and two as E/L genes by our PAA analysis. The only exception was ul13 which although an L gene in the HSV, behaved as an E gene in all of our analyses. The profile curves of the genes clustered on the basis of Pearson's correlation of pairwise R_Δ _values, gave very similar patterns of expression within the groups. In each period of time, the expression curves can also be characterized by the formula: R_Δ__(t+1) _- R_Δ__t _(Table 2). For example, group L1 was characterized by hardly any change in the first 2 h, a moderate rise in mRNA level between 2 and 4 h, and a sudden increase in mRNA copy number from 4 to 6 h (0/+/++). Group L2 (r ≥ 0.99) contains one E/L and 6 L genes. The expression profiles of these genes are characterized by a continuous intensive increase from 2 to 6 h (0/+/+). Our PAA analysis revealed that all of the 6 members of group L3 (r ≥ 0.99) are L genes, except for *us1*, which is an IE gene in the HSV. In our system, therefore, most of the data (except the relatively high R_2h _for *us1*), suggest that it is an L gene. This group is characterized by the expression profile 0+/0-/+++. Group E1 (r ≥ 0.91; with 5 members) and group E2 (r ≥ 0.901, with 5 members) comprise E genes, except for *ul53*, which is an L gene in the HSV. However, this gene is characterized as an E gene in the PRV [39], which was confirmed by our PAA analysis. These genes are characterized by the expression profile: 0+/+/-. Group E3 (r = 0,964, 2 members: *ul11 *and *ul9*), expression profile: +/+/0+; Group E4 (r = 0.996, 2 members: *ul12 *and *us4*), profile: 0/+/0. Group M1 (r ≥ 0.933) is composed of 8 members, shown by our PAA analysis to exhibit either E or L kinetics. The expression profiles of these genes are very similar (0/+/++), which draws attention to the fact that PAA analysis alone is not sufficient for the grouping of genes with a similar expression profile. The 3 members of group M2 exhibit a very high correlation (r ≥ 0.998) and similar expression profiles (0/++/+). Group LLT (r > 0.946) contains 2 *llt *genes that have an unusual expression profile (++/--/+++). The following 4 genes cannot be clustered on the basis of Pearson's coefficients: *ie180 *(--/++/++), *ul30 *(--/--/++), *ul34 *(+/+/+) and *us3 *(+/++/---), which are likewise unusual Indeed, these genes have peculiar expression profiles (Figure 16). The expression profile can also be visualized by heatmap presentation, using the expression data generated with the R_Δ__t_values (Figure 17).

**Table 2:** Grouping PRV genes on the basis of expression curves

**group**	**L1**	**L2**	**L3**	**E1**	**E2**	**E3**	**E4**	**M1**	**M2**	**LLT**
**profile**	**0/+/+++**	**0/+/+**	**0/0/+++**	**0/+/-**	**0/++/--**	**+/+/0+**	**0/+/0**	**0/+/++**	**0/++/+**	**++/--/+++**
gene	*ie180*	*ul30*	*ul34*	*us3*						
profile	**--/++/++**	**--/--/++**	**+/+/+**	**+/++/---**						

Gene expression profiles of PRV gene clusters were characterized by plus and minus signs at various time intervals [R_t(2h-1h_) - R_(t1h-0h) _; R_t(4h-2h_) - R_(t2h-1h)_; R_t(6h-4h_) - R_(41h-2h)_]. The numbers of plus or minus signs indicate the relative rate of increase or decrease of transcripts.

### Co-regulated gene clusters

We utilized our data to analyze whether the expression properties of PRV genes localized in adjacent genomic loci display similarities.

#### PAA analysis

**Figure 18:**
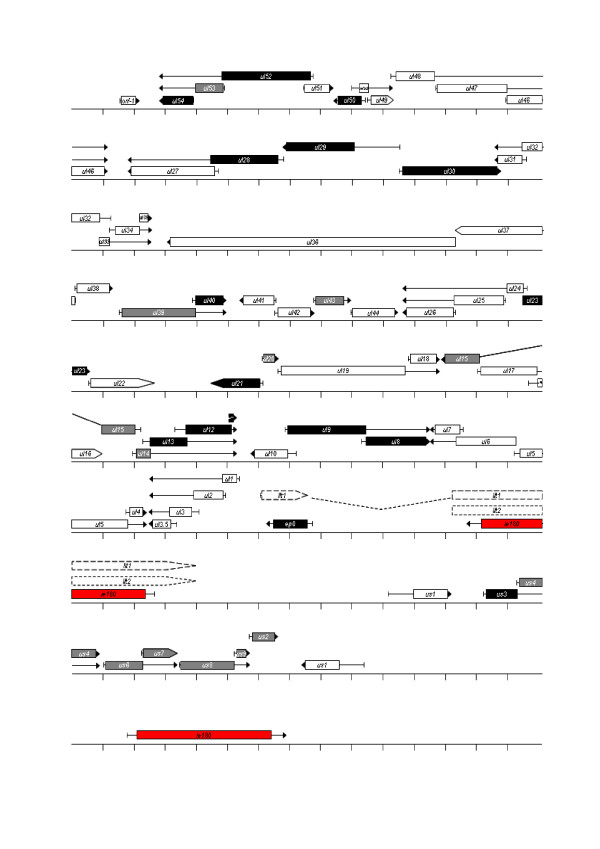
**PRV genome**. PRV genes are represented as arrows, which indicate their relative lengths, orientations and kinetic classes determined by PAA analysis. Red indicates IE kinetics, black E kinetics, gray E/L kinetics, and white L kinetics.

Figure 18 shows an intriguing relationship between genes categorized on the basis of the data of the PAA experiments. Nested 3' co-terminal genes were found to belong in the same kinetic classes, indicating the existence of shared regulatory mechanisms of gene expression. An exception is the late *ul27 *gene, which forms a cluster overlapping in parallel with the *ul28 *and *ul29 *genes expressed with E kinetics. Of the 11 divergent pairs, 6 genes were found to belong in the same and 5 in different kinetic classes. A further special feature of the PRV genome is that the convergent genes mostly belong in different kinetic classes (Figure 18). The potential role of such organization of the herpesvirus genome is discussed later. It may be noted that most US genes belong in the E/L kinetic class accounting for 37.5% of this group.

#### Pearson's correlation

**Figure 19:**
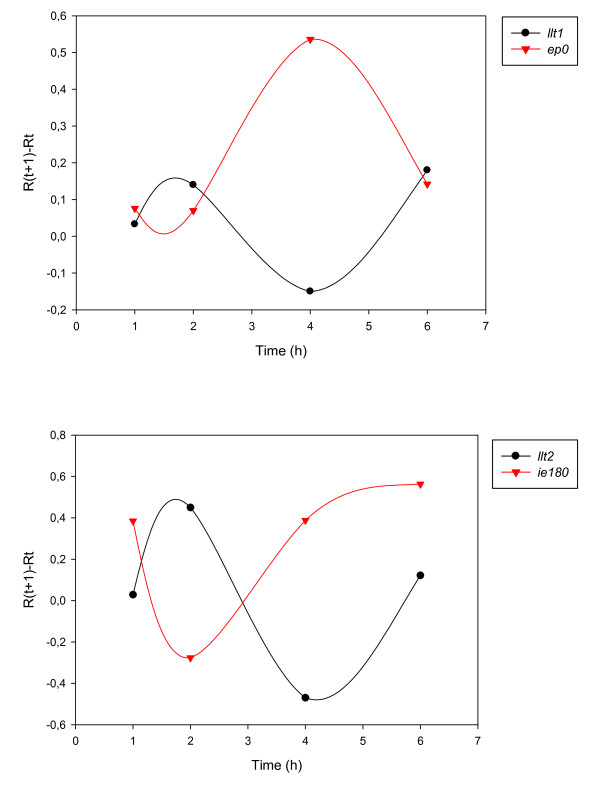
**Inverse expression profiles of sense/antisense transcripts**. The expression curves of R_Δ _reveal show an inverse relationship between the mRNAs of genes and their antisense transcripts: *ep0*/*llt1 *and *ie180*/*llt2*.

Analysis of the gene clusters generated by using Pearson's correlation coefficients revealed that genes within the same clusters are often located in neighboring genomic positions. Group L1 is composed of 24 members. The adjacent *ul1*, *ul2*, *ul3*, *ul5 *and *ul6 *genes are members of this group. The high pairwise correlation coefficients and the similar profile curves of the R_Δ _values of these genes cannot be explained by the nested co-localization alone, because *ul5 *and *ul6 *are divergent genes and do not overlap with *ul1*, *ul2 *and *ul3*. The *ul44 *gene is convergent with *ul24 *and *ul26*; and *ul22 *is divergent with *ul20*. Moreover, *ul27 *is convergent with *ul47 *and *ul48*. It should e borne in mind that *ul26 *and *ul27*, an adjacent gene pair in the HSV, are relocalized into a remote position in the PRV, due to a large inversion, but retain their expression properties. This is also true for *ul44, ul47*, and *ul48 *(there is no *ul45 *gene in the PRV). Several genes (*us2*, *us6*, *us8 *and *us9*) in the US region of the PRV genome belong in this group, too. The *ul17 *and *ul19 *genes, which are oriented parallelly, but non-overlapping, likewise belong in group L1. The *ul34 *and *ul35 *genes are in a divergent position with *ul37*. Group L2 is composed of 7 members. The *ul32 *and *ul33 *genes are divergently oriented, while *ul4 *and *ul3.5 *are convergent genes. Group L3 is a small group with 6 members. The *ul36 *and *ul38 *genes are arranged in a divergent orientation. Group E2: Two of the 4 members of this group, *ul28 *and *ul29*, are overlapping genes. Group E3: Two (*ul11 *and *ul12*) of the 4 members of this group are divergently oriented with the third member, *ul9*. Group M1: *ul7 *and *ul8 *are a convergent gene pair. The *ul49.5 *and *ul51 *are L genes oriented in parallel and separated by the divergent early *ul50 *gene. Furthermore, *ul15 *and *ul17 *are a convergent adjacent gene pair. Genes with similar expression profiles appear to be under a common regulation, which cannot be explained solely by the nested localization (these genes also have common promoters) of genes. Group LLT: the *llt1 *and *llt2 *expression patterns show a high degree of similarity. However, there is noteworthy inverse relationship in the expression profiles of LLT1 and LLT2 with those of their complementary partners, EP0 and IE180 mRNAs, respectively (Figure 19).

## Discussion

Gene expressions of α-herpesviruses have already been analyzed by various techniques including Northern-blot and microarray analysis. Different authors have often assigned the same genes to different kinetic groups, which may be explained by the poor resolution of the applied techniques. Only partial data are available on the expression of PRV genes. Flori *et al*. [27] carried out genome-wide gene expression analysis via a microarray technique, but this approach did not produce evaluable data on the kinetic properties of the PRV genes. We employed a novel qRT2-PCR technique using (1) strand-specific primers for the RT to obtain a higher yield and to eliminate the effects of the potential antisense transcripts; and (2) the average maximal E^Ct ^values as the controls for the calculation of relative expression ratios (Rs). Other approaches, such as the models of Pfaffl's [62] and Souazé et al., [63], and the ΔCt and ΔΔCt models [64] are simpler than the model we used; however, they all neglect the variation in the amplification efficiencies between cDNA samples, which was present in our system. Traditionally, herpesvirus genes are grouped into kinetic classes on the basis of gene expression inhibitory effects of protein and DNA synthesis-blocking reagents. The IE genes should not be significantly inhibited by any of the above substances, the E genes are inhibited only by DNA synthesis blockers, and the expression of the L genes is substantially inhibited by both protein and DNA synthesis blockade. We grouped the PRV genes into IE, E, E/L and L categories on the basis of the results of CHX and PAA treatment (Table 1), [see Additional file 3]. Our findings conformed well with those published for the HSV genes. The expression of the *ie180 *gene is enhanced by CHX treatment. The explanation of this phenomenon lies in the fact that the *ie180 *protein normally inhibits its own expression a short time after the onset of virus infection, a feature which is absent in CHX-treated cells due to novel protein synthesis being blocked by the drug. The other PRV genes were inhibited to a significant extent by CHX at every examined time point. Consequently, *ie180 *is the only IE gene of the PRV. The *ep0 *and *ul54 *genes (IE genes in the HSV) were shown to be E genes by others [[10] and [11]], and this was confirmed by our analyses. Interestingly, *us1*, which is an IE gene in the HSV, appeared to be an L gene in most of our analyses: its expression was significantly inhibited by PAA; it afforded a low amount of mRNAs at 1 h pi, and a high rate of increase of transcript level at 4-6 h pi, all of these being L characteristics. Furthermore, together with a set of L genes, *us1 *belongs in group L2 according to the clustering of the genes on the basis of pairwise Pearson correlation coefficients. The moderately high relative amount of *us1 *transcripts at 2 h pi is the only E-like characteristic of this gene. However, *us1 *displays a high Ct value, suggesting a low absolute amount of transcripts at 2 h pi. We may mention here that the Ct value of a transcript is dependent on the primer efficiency; these data therefore cannot be reliably used to the estimate the absolute transcript amount. If the *us1 *gene has an important function early in the lytic cycle of the virus, it might fulfill this without *de novo *synthesis by being released upon infection from the tegument layer of the virus where it might be incorporated. Our data do not demonstrate a clear-cut demarcation line between the groups of E and L genes, suggesting that the categorization of herpesvirus genes on the basis of the requirements for DNA replication might be arbitrary. The kinetic types of genes identified by our PAA analysis resemble homologous HSV genes more than the published PRV genes, which can be explained by the semiquantitative methods used for the kinetic analysis of the PRV genes. The present study revealed that temporal classification of herpesvirus genes is also possible through study of the gene expression of untreated cells in consequence of the high sensitivity of the real-time RT-PCR technique. As a further interesting point, we detected mRNAs by qRT2-PCR in all but 3 PRV genes as early as 1 h pi [see Additional file 4]. This observation indicates that PRV genes are either leaky or subject to regulatory mechanisms which have not yet been elucidated. We found that E genes are generally expressed in a higher proportion than L genes at 1, 2 and 4 h, as indicated by their higher R values at these time points [see Additional file 4a]. The E gene products proved to display a higher net increase (R_Δ_) than the L genes within the 0-1 h, 1-2 h and 2-4 h pi periods. However, by the 4-6 h pi interval, this trend had definitely reversed, i.e. the L genes exhibited high R_(6h-4h) _values (Additional file 4b). Unexpectedly, the ratio of increase (R_a_) between 1 and 2 h was higher for a number of L genes than any of the E genes [see Additional file 4c]. Since the R_2h _values of these L genes were lower than those of the E genes, the high R_(2h/1h) _value is a result of the very low R_1h _values of late genes. Most of the L genes also have higher R_(4h/2h) _and R_(6h/4h) _values than those of E genes, which is explained by the fact that the E genes reach a relatively high expression level by 1 h pi. The classification of the PRV genes through the CHX and PAA analyses led to results very similar those generated by the analysis of untreated cells via the following calculation: R_(6h-4h)_/R_(1h-0h) _[see Additional file 5]. The differences between the results of the two approaches can be explained in that PAA analysis alone gives only a rough picture of the gene expression; a detailed kinetic analysis of the viral gene expression furnishes a more sophisticated picture. Some genes classified as E or L by PAA analysis display "irregular" kinetics under untreated conditions. The *ie180 *and perhaps *us1 *genes cannot be classified by PAA analysis alone. Analysis of the transcription kinetics of overlapping transcript sets is an important issue. It is not known whether downstream genes on polycistronic mRNAs are translated, and if so, to what extent. This makes interpretation of the mRNA expression data difficult because the mRNA levels cannot be correlated with the amounts of the corresponding proteins, which are the workhorse molecules in regulating cellular physiology. In principle, a downstream gene is translated if it is transcribed from its own promoter. Theoretically, downstream genes could also be translated from a polycistronic RNA if alternative splicing removed upstream gene(s) from the pre-mRNA, or if potential IRES-like sequences helped recruit ribosomes, thereby initiating a cap-independent translation from the downstream gene (we have found no data in the literature concerning these possibilities). Thus, analysis of the downstream genes without discriminating between transcription from their own promoters or by read-through from upstream genes might result in a false categorization. The PRV genes were also classified by analysis of the gene expression throughout the entire examination period by using Pearson's correlation analysis. Genes with similar expression profiles (high pairwise r values) were placed in the same group. Genes in the same kinetic class (identified by PAA analysis) are generally situated in the same group generated by the use of Person's correlation. Moreover, members of a particular group (with high r values) rarely display high correlation coefficients with genes belonging in different groups. The PRV genes conformed well to 10 patterns of expression. Only 4 viral genes (*ie180*, *ul30*, ul34*and us3*) did not fit into any of the 10 groups. We found an inverse expression profile of the sense/antisense partners in the *ep0*-*llt1 *and *ie180*-*llt2 *genes (Figure 19). Combination of the untreated data with the results of the CHX and PAA analyses revealed an interesting relationship for coordinated regulation of sense/antisense partners. The CHX analysis suggested that IE180 protein facilitates LAP (latency-associated transcript promoter) activity, and inhibits ASP (putative antisense promoter activity). The PAA treatment resulted in a significantly elevated antisense transcript level at 3 of the 4 time points in LLTs, which indicates the existence of another regulatory level besides IE180 protein action. We assume that transcription from one DNA strand negatively influences the expression of transcripts from the complementary DNA strand. The interaction can occur at the level of transcription (RNA polymerase moving in one direction along one of the DNA strands inhibits RNA polymerase moving in another direction) and/or translation by forming double-stranded RNAs by the sense and antisense transcripts. As an example, PAA has a negative effect on the transcription of *ie180 *(the level of IE180 mRNA is reduced to a quarter) at 4 h pi, which results in a lower rate of transcription from *ie180 *(thereby facilitating the expression of LLT2); and a lower amount of inhibitory IE180 proteins, which also facilitates LLT2 expression. Overall, the LLT2 level increases 39.4-fold relative to the untreated conditions. Furthermore, genes with the same kinetic properties exhibit a distinctive distribution pattern along the PRV genome. Nested genes appear to belong in the same kinetic group. Additionally, convergent genes and gene clusters in most (3 out of 11) cases belong to different kinetic classes. It should be remembered that the PAA-based approach and the time intervals applied for kinetic analysis produce only a coarse resolution of gene expression. It is possible that convergent genes allocated to the same kinetic classes might display different expression profiles on a finer scale. The above genome organization principles may point to the existence of as yet unknown regulatory mechanisms. It may be speculated whether a read-through of transcription across convergent genes is the basis of this regulation. Moreover, groups formed in terms of high Pearson's correlation coefficients contain many genes localized at adjacent loci on the PRV genome. Most genes with high correlation coefficients are not nested genes. This means that the similarity of their expression profiles cannot be explained by the control of a common promoter. Further, several genes in the same group are separated by genes that display different kinetic profiles. These results suggest the existence of a genetic mechanism that synchronizes gene expression on a higher-order (chromatin?) scale.

## Conclusion

Although the relative amounts of the transcripts between genes cannot be compared because primer efficiencies may vary both in RT and in PCR, our method allows a comparison of genes on the basis of their expression dynamics. We detected the expression of almost all genes from the earliest time point (1 h), Further, we have found that a sharp boundary cannot be drawn a between the groups of E and L genes in terms of expression profiles. We have also found that the PRV has only a single IE gene (the *ie180 *gene) and that the *us1 *gene is expressed in L kinetics. Our kinetic studies revealed that PAA analysis alone is not sufficient for the classification of genes on the basis of their expression profiles. The analysis of gene expression throughout the 6 h examination period demonstrated that these genes can be subdivided into further clusters, and that genes showing significant differences in response to PAA treatment can have very similar expression characteristics in untreated conditions. In our analysis, nested genes displayed the same expression profiles, whereas convergent genes mostly exhibited different kinetic properties. It should be noted that a finer resolution of the gene expression might expose slight differences in the expressions of nested genes. Approximately half of the divergent genes belong in the same class, and the other half of them in different kinetic classes. The new calculation technique that we have developed is also applicable to evaluate the loss-of-function phenotypes of mutant herpesviruses viruses and, in principle, for the analysis of gene expression in every temporally changing genetic system.

## Methods

### Cells, viruses and infection conditions

Monolayer cultures of immortalized porcine kidney 15 (PK-15) cells were maintained at 37°C in an atmosphere of 95% air, 5% CO_2 _in Dulbecco's modified Eagle medium (DMEM) supplemented with 5% fetal bovine serum (Gibco) and 80 *μ*g gentamicin per ml. Strain Kaplan of pseudorabies virus (PRV-Ka) was used for gene expression analysis. The virus stock was prepared by infecting PK-15 cells with 10 plaque forming units (pfu)/cell PRV-Ka followed by incubation of the cells until a complete cytopathic effect was observed. For the analysis of the PRV transcriptome, rapidly-growing semi-confluent PK-15 cells were infected with a low multiplicity of infection (MOI; 0.1 pfu/cell) of the virus, and incubated for 1 h, after which the virus suspension was removed and the cells were washed with phosphate-buffered saline (PBS). Subsequently, new culture medium was added to the cells, which were further cultivated for 0, 1, 2, 4 or 6 h. Cells were incubated in the presence or absence of 100 *μ*g/ml cycloheximide (CHX), a translation inhibitor, or 400 μg/ml phosphonoacetic acid (PAA), an inhibitor of DNA synthesis (both purchased from Sigma-Aldrich) 1 h prior to virus infection. Mock-infected cells, treated in the same way as infected cells, were used as controls.

### Preparation of viral DNA

Viral DNA was used to test the efficiency and specificity of the primers applied in real-time PCR. The viral DNA was purified as follows. Monolayers of PK-15 cells were infected with the PRV at an MOI of 10, and cultivated at 37°C until a complete cytopathic effect was observed. Subsequently, culture medium was collected without disrupting the cells and clarified by centrifugation at 4,000 rpm for 10 min using a Sorvall GS-3 rotor. Next, the virus in the supernatant fluids was sedimented on a 30% sucrose cushion by ultracentrifugation at 24,000 rpm for 1 h using a Sorvall AH-628 rotor. The sedimented virus was resuspended in sodium Tris-EDTA buffer. After the addition of proteinase-K (l00 μg/ml final concentration) and sodium dodecyl sulfate (SDS; 0.5% final concentration), the lysate was incubated at 37°C for 1 h, which was followed by phenol-chloroform extraction and dialysis.

### Primers

Primers were designed through use of the Primer Express (Applied Biosystems) and FastPCR Professional (Primer Digital Ltd.) oligonucleotide design software according to the given guidelines. Primer pairs were designed from the 3'-end regions of ORFs for each gene (Table 3). The *ul26 *and *ul26.5*, and *orf1 *and *orf1.2 *genes contain overlapping ORFs; and we therefore, did not employ distinct primers for them. Primer specificity was verified by BLAST searches of the GenBank database (National Center for Biotechnology Information (NCBI) website . All primers were purchased from Bio Basic Inc. (Mississauga, Ontario, Canada).

**Table 3:** Primer sequences^a^

**Name^b^**	**Forward primer sequence (5'-3')^c^**									**Reverse primer sequence (5'-3')^d^**								**Primer coordinates^e^**	**Size of amplicon (bp)^f^**
***orf1***	GCC	TCG	TGT	TTC	TGA	TCC	TGC	TC		AAC	ACG	ACA	GAC	TCC	CGG	AGC	A	2204-2252	49
***ul54***	TGC	AGC	TAC	ACC	CTC	GTC	C			TCA	AAA	CAG	GTG	GTT	GCA	GTA	AA	2730-2795	66
***ul53***	CCA	AGG	CGC	TGT	ACC	TCT	G			TGT	GCC	GCT	CAT	AGT	GCA	G		3912-3977	66
***ul52***	CGC	GCA	ACT	TTC	ACT	TCC	ACG	CA		TGC	GCT	CGA	AGA	AGC	TCT	CGT	A	5325-5376	93
***ul51***	GCT	CAT	GCA	CCT	GTA	CCT	CTC	G		ACG	TCG	GAC	ATC	ACC	ACG	TTG	C	7989-8096	108
***ul50***	CTT	CTT	CGA	GGT	CTT	TGC	GC			ATG	TCG	TAT	CCG	GCG	TCC	T		8921-8971	51
***ul49.5***	TGA	CAT	TTT	ATA	TCT	GCC	TCC	TGG		TGC	AGC	ATC	CGG	GTG	C			9447-9525	79
***ul49***	CAT	CAC	CGT	GTG	CGA	G				CTT	TTC	CCT	TCC	GCC	CG			10247-10329	83
***ul48***	AGG	TGC	GGA	TCA	AGA	TGG	AG			CAG	TAC	GTG	TGG	TCG	CGG	T		11305-11359	55
***ul47***	GAG	CTC	ATG	GAC	GCG	CTC				CTG	GCG	CAT	GAC	GGC	GTC			13684-13767	84
***ul46***	TGC	TGT	GGT	CGA	CGT	TCG				TCT	GGG	CCT	TGT	GCT	TGA	A		14435-14494	60
***ul27***	ACT	ACG	AGG	ACT	ACA	ACT	ACG	TGC	G	GTC	ACC	CGC	GTG	CTG	ATC			17512-17575	64
***ul28***	GCG	CTC	TAC	TTT	GCG	GTG	GAG	AA		GAA	CTC	GCT	GAC	GCA	CCA	ATC	G	19781-19879	117
***ul29***	CTG	ATC	CTG	CGC	TAC	TGC	G			ACT	GCA	TCG	TGA	TCC	CCG			22609-22675	67
***ul30***	TCA	TCA	CGA	AGA	AGA	AGT	ACA	TCG	G	CCT	TCA	TGA	GCA	TCT	TGC	CG		27959-28015	57
***ul31***	GTG	GCA	GAC	CAT	GTT	CGT	GT			GGT	CTC	CCG	TCT	CCC	TTC	TT		28809-28859	51
***ul32***	TGC	TCA	GCT	ACT	CGG	AGA	ACA	AC		CAC	GGG	CTC	GAT	GCA	GTC			29841-29893	53
***ul33***	CGC	GCG	AGC	TGG	AAG	T				TGC	GTG	TGG	GCC	AGA	TAA			31031-31094	64
***ul34***	AGC	ACA	ACA	ACG	TGA	TCC	TGG			GAG	AGG	CTC	ATG	CCG	GTG			31696-31747	52
***ul35***	ATC	ATG	TCC	TTC	GAC	CCG	AAC			GAG	CGT	CTG	CGC	GGT	GA			32238-32288	51
***ul36***	CGT	CGG	TGG	GTA	TTA	GAG	ACC	A		GAA	CAA	GAG	CCA	TGG	ATT	TTC	G	34451-34501	51
***ul37***	CTA	CGA	CAT	GGA	CTT	TGT	GCA	GGA		AGT	TGG	TGT	GCT	GCG	CCA	CGT	A	44556-44611	56
***ul38***	CAC	CCG	GAA	CTC	GTG	CAT	GTA			CAG	AAG	AAA	CAC	TCC	TCC	CAC	G	46118-46273	156
***ul39***	GCT	GGC	CAA	GTT	CAA	GAC	G			CGC	ACA	TGT	CGA	TGA	GCA	G		48701-48759	59
***ul40***	GGA	CTT	TCC	CAT	GGC	GC				GCT	CGA	AAA	AGT	TGG	TGT	GCT	T	49802-49854	53
***ul41***	TGA	AGA	ACG	AGA	CGC	GGG				TGT	GTT	TCC	AAA	ACA	GGC	CC		50475-50525	51
***ul42***	GCT	CCC	CGA	GCG	TCG					CAT	GAT	GCA	GTA	GTC	GTT	GAA	CTC	52118-52173	56
***ul43***	CTG	GTG	CAG	GCG	TAC	GTG	A			GGA	TTT	AAT	GCT	AGT	GGC	GCA		53904-53954	51
***ul44***	TCG	TGA	GCA	GCA	TGA	TCG	T			GTC	GCC	ATG	ATG	ACC	AGC			55368-55437	70
***ul26***	TTC	TTC	CTC	GGC	GTC	GTC	AAC	TG		GTA	GTT	GCT	CAG	CAG	GTA	CAG	CA	56962-57087	126
***ul25***	GGC	AGT	TTG	GCG	TCT	CCA	G			CCA	GGC	AGA	GAA	AGT	ACA	GGA	GG	57336-57386	51
***ul24***	TGT	GCT	TCG	TCA	TCG	AGC	TC			TGG	GCG	TGT	TGA	GGT	TCC			59249-59305	57
***ul23***	ATG	ACG	GTC	GTC	TTT	GAC	CGC	CAC		CGC	TGA	TGT	CCC	CGA	CGA	TGA	A	59818-59898	81
***ul22***	GGC	GGC	CAT	CAC	CGT					GAG	AAT	AGC	CCT	CGG	AGG	AGA		62574-62647	74
***ul21***	TCA	GCT	GTT	TCG	GGC	GC				ATT	GAG	GAC	GAT	GGA	GAT	GTT	GG	64500-64551	52
***ul20***	GAG	AAC	GAC	GCG	CTG	CTG	AG			CAG	GAG	GCT	CAC	CAC	GTG			66226-66309	84
***ul19***	TCT	TTG	CGG	AGA	AGG	CCA	G			GCT	CTC	GGT	GCG	CGC				69841-69905	65
***ul18***	TGG	TGC	TGA	ACA	TGA	TCT	TCC			GGA	TGA	GCG	ACA	GCA	GGA	T		71443-71502	60
***ul17***	GGC	GTT	TTC	CTC	TTC	GAC	TAC	TAC		CAC	CCT	TAT	AAC	CTC	CCC	GC		74663-74713	51
***ul16***	CTG	TGG	CGC	CAG	GCG	GAC	AA			GAT	CTT	GCG	GCG	GGG	GAG	CAT	G	75670-75780	111
***ul15***	CAG	AAG	CAA	AAG	ACC	CCC	G			CGA	GTT	GAA	CTG	CTT	GAC	GAA	A	72204-72254	51
***ul14***	CGG	ACA	AGA	AAA	ACC	CCG	AG			CCT	GTT	TGG	CCG	CCA	TAA	A		77167-77217	51
***ul13***	AGC	CAC	CTG	GAC	GTC	AAG	G			CCA	TGA	GGC	TAA	AGT	CCC	CG		78083-78173	91
***ul12***	GCA	GAC	GGA	GAT	GCG	CTT				CCG	AGA	ACA	GGT	ACT	TGG	CG		79394-79449	56
***ul11***	ATG	GGA	CAG	TGT	TGC	TGC	C			TCA	AAG	TCC	TCG	AAC	GCG	T		80084-80178	95
***ul10***	CAT	TTT	GTG	TTT	CTC	GCC	CTC	TTT		ACC	CGT	GCC	CTT	GCA	GG			81390-81443	54
***ul9***	CAA	GTT	CAA	GCA	CCT	GTT	CGA			TGA	GGC	TGT	CGT	TGA	CGC			83337-83392	56
***ul8***	CCG	CTG	ATC	CTG	CCC	TG				GAA	GAT	GGG	CTC	CAT	GTG	G		86355-86405	51
***ul7***	TCC	GCG	GGT	TCG	CCT	TC				AGC	GAG	AGC	ACG	CGG	TC			87217-87271	55
***ul6***	CAG	GAG	CTG	ATC	CGC	TGC				TGT	TGG	AGT	ACG	AGA	CGG	ACA	C	87687-87765	79
***ul5***	TGG	ACA	TGG	CCA	CCT	ACG	T			ACC	GCG	CGA	TGG	TCA	T			91530-91592	63
***ul4***	GAG	CGA	GAC	CGA	CCA	TGA	C			TTA	TTT	CGT	AAA	ACG	AGT	AGG	CCA	91849-91899	51
***ul3.5***	CAA	CGG	AGC	ATC	AAC	GCC	TC			ATT	GAT	CGC	GGT	AGC	AAC	AGG	A	92478-92658	181
***ul3***	CTG	GGC	GCA	GCA	CGT					TCC	ACG	GAC	GCG	ACC	ATA			93502-93552	51
***ul2***	TTC	AGG	ACC	TGC	CCG	CAC	TTT	GGA		ACT	CAG	TCC	ACG	CTC	CAG	TCG	A	93914-93999	86
***ul1***	CTG	GTC	AAC	CCC	TTT	GTC	G			CGC	CTC	ATT	TAA	GGG	CTC	TC		94928-94987	60
***ep0***	GGG	TGT	GAA	CTA	TAT	CGA	CAC	GTC		TCA	GAG	TCA	GAG	TGT	GCC	TCG		96956-97006	51
***ie180***	CAT	CGT	GCT	GGA	CAC	CAT	CGA	G		ACG	TAG	ACG	TGG	TAG	TCC	CCC	A	103988-104056	69
***us1***	AGC	TCA	ACG	AGC	GCG	ACG	TCT	A		CGG	AAG	CTA	AAC	TCG	GAC	GCG	A	116466-116602	137
***us3***	GGG	CTT	TCC	TGA	TTT	ACA	AGA	TGT		AAG	GGC	GGC	GGA	CG				119245-119295	51
***us4***	ACC	TCG	ATC	TAC	ATC	TGC	GTC	G		GGC	CCT	GGT	GAT	CGC	CAT			120764-120850	87
***us6***	TGG	AAC	GAC	GAG	AGC	TTC	AGG			GTA	GAA	CGG	CGT	CAG	GAA	TCG		121690-121749	60
***us7***	CCC	GGG	AAG	ATA	GCC	ATG				AAG	AAG	ATC	AGG	AGG	ACG	ACG		123144-123199	56
***us8***	CTT	CGA	CGT	CTG	GTT	CCG	C			GGT	CAC	GCC	ATA	GTT	GGG	C		125136-125202	67
***us9***	CAG	GAC	GAC	TCG	GAC	TGC	TA			AGG	AAC	TCG	CTG	GGC	GT			125395-125453	59
***us2***	CGG	CTG	GAC	ACG	GAG	TG				AGT	TCA	GGT	ACT	GGA	TCC	CGT	T	126171-126222	52
***28S***	GGG	CCG	AAA	CGA	TCT	CAA	CC			GCC	GGG	CTT	CTT	ACC	CAT	T			54

^a ^This table shows the list of primer pairs used for analysis of PRV gene expression:

^b ^name of the primers

^c^forward primer sequences

^d^reverse primer sequences

^e ^Coordinates refer to the PRV genome sequence in strain Ka (NCBI Reference Sequence: NC_006151.1).

^f^size of the amplified products (amplicons) Underlined sequences were not available from strain Ka, and therefore we sequenced these DNA segments.

### Polymerase chain reaction

A conventional PCR technique was used to check on the potential DNA contamination and the quality and specificity of the primers. The PCR reaction was carried out by standard methods on a Veriti™ 96-Well Thermal Cycler (Applied Biosystems) with the following parameters: 1 cycle of 94°C for 4 min; 30 cycles of 94°C for 1 min, 60°C for 1 min and 72°C for 2 min; and 1 cycle of 72°C for 7 min. The PCR reaction was carried out by using the GC Rich PCR System (Roche). The complete DNA sequence of the PRV is a composite of 6 different strains [65], but the variation between them is very small (around 1%). Most of the sequence data (86.7%) have been derived from strain Kaplan (PRV-Ka), which we used in our experiments. To circumvent the problem of genetic background differences, we tested 2 or more primers for RT and PCR reactions for non-Ka sequences and selected those that performed best; alternatively, we sequenced the particular DNA region and designed new primers on the basis of the sequence data. For each gene, we designed and tested various primers, and selected those, which did not produce primer dimers or other nonspecific products. For those genes where we could not eliminate the problem of primer dimer formation, in every cycle we applied an extra extension step with an elevated temperature (below the Tm of the specific product and well above the Tm of the primer dimers) for the detection.

### RNA preparation

PK-15 cells (5 × 10^6 ^cells per flask) were washed in PBS and harvested for RNA isolation at 0, 1, 2, 4 and 6 h pi. For quantitative RT2-PCR, total RNA was extracted from the cells with the NucleoSpin RNA II Kit (Macherey-Nagel GmbH and Co. KG) according to the manufacturer's instructions. Briefly, cells were collected by low-speed centrifugation, lysed in a buffer containing the chaotropic ions needed for the inactivation of RNases and providing the conditions for binding of nucleic acids to a silica membrane. Next, contaminating DNA was removed with RNase-free rDNase solution (included in the NucleoSpin RNA II Kit). Subsequently, the RNA solution was treated with Turbo DNase (Ambion Inc.) to remove potential residual DNA contamination. Finally, RNA was eluted in RNase-Free Water (supplied with the kit) in a total volume of 60 μl. RNA concentration was determined in triplicate by spectrophotometric analysis of the absorbance at 260 nm with a BioPhotometer Plus (Eppendorf). The RNA solution was stored at -80°C until use.

### Quantitative real-time RT-PCR

A two-step quantitative real-time RT-PCR was carried out for the transcriptional analysis. For each gene, a minimum of 3 independent replicates (separate infections) were performed.

*Reverse transcription (RT) *Total RNA isolated from infected cell cultures subjected to drug treatment (CHX or PAA) was reverse-transcribed into cDNA for PCR analysis. RTs were performed in 5 *μ*l of solution containing 0.07 *μ*g of total RNA, 2 pmol of the gene-specific primer, 0.25 *μ*l of dNTP mix (10 *μ*M final concentration), 1 *μ*l of 5× First-Strand Buffer, 0.25 *μ*l (50 units/*μ*l) of SuperScript III Reverse Transcriptase (Invitrogen) and 1 U of RNAsin (Applied Biosystems Inc.) and the mixture was incubated at 55°C for 60 min. The reaction was stopped by raising the temperature to 70°C for 15 min. No-RT control reactions (RT reactions without Superscript III enzyme) were run to test the potential viral DNA contamination by conventional PCR. RNA samples with no detectable DNA contamination were used for quantitative RT-PCR reactions. First-strand cDNAs were diluted 10-fold with DEPC-treated water (Ambion Inc.), then subjected to real-time PCR analysis.

*Real-time quantitative PCR *experiments were performed with a Rotor-Gene 6000 cycler (Corbett Life Science). All reactions were carried out in 20-*μ*l reaction mixtures containing 7 *μ*l of cDNAs, 10 *μ*l of ABsolute QPCR SYBR Green Mix (Thermo Fisher Scientific), 1.5 *μ*l of forward and 1.5 μl of reverse primers (10 *μ*M each). The running conditions were as follows: (1) 15 min at 95°C, followed by 30 cycles of 94°C for 25 sec (denaturation), 60°C for 25 s (annealing), and 72°C for 6 s (extension). The absence of nonspecific products or primer dimers was indicated by observation of a single melting peak in melting curve analysis. An additional extension and detection step was applied for those primers that produced primer dimmers: for 2 s at a temperature just below the Tm of the specific product and substantially above the Tm of the primer dimers. With this technique we could eliminate nonspecific fluorescent signals produced by primer dimers. Following the PCR reaction, melting curve analysis was performed to control amplification specificity (specificity was defined as the production of a single peak at the predicted temperature and the absence of primer dimers) by measuring the fluorescence intensity across the temperature interval from 55°C to 95°C. The 28S ribosomal (r)RNA used as the loading control (reference gene) was amplified in each run. H_2_O was included as a no-template control, and cDNA derived from the reverse-transcribed RNAs of non-infected cells was used as a negative mock-infected control. We applied SYBR Green-based real-time PCR because of the lower costs and simpler protocol than for TaqMan probe-based methods for instance. It has recently been demonstrated that the SYBR-based method of detection is as sensitive and specific, and has a similar dynamic range to that of the TaqMan-based technique [66].

### Calculation of relative expression ratio (R)

We calculated the R value by using the following equation:



where R is the relative expression (quantification) ratio; E is the efficiency of amplification; Ct is the cycle threshold value; sample refers to any particular gene at a given time point; and ref is the 28S rRNA, which was used as a reference gene and was amplified in each run. Average Ct values with their standard error (SE) values and amplification efficiencies with SE are shown in Additional file 6. This equation is similar to that used by Soong *et al *[30]. However, instead of individual values, we used the average maximal value of E^Ct ^for each gene as the control. The relative copy numbers of mRNAs were calculated by normalizing cDNAs to 28S rRNA using the Comparative Quantitation module of the Rotor-Gene 6000 software (Version 1.7.28, Corbett Research), which automatically calculates the real-time PCR efficiency sample-by-sample. Thresholds were set automatically by the software.

### Analysis and presentation of data

Data were analyzed by the Microsoft Excel program, using the average and the standard deviance functions. The inhibitory effect of CHX or PAA was calculated via the ratio of the drug-treated and untreated R values at 2, 4 and 6 h pi for CHX: R_i-CHX = _R_CHX_/R_UT_, or 4 and 6 h pi for PAA: R_i-PAA = _R_PAA_/R_UT_. Thus, a low value indicates a high inhibitory effect and *vice versa*. The net increase in a product was calculated by subtracting the R value at time point t+1 from that at t (R_Δ _= R_(t+1)_-R_t_), where t = 0, 1, 2, 4 or 6 h and (t+1) = 1, 2, 4 or 6 h. The ratios of adjacent R values (rate of change; R_a_) were calculated with the following equation: R_a _= R(_t+1_)/R_t_; t = 1, 2, 4 or 6 h; (t+1) = 2, 4 or 6 h. Pearson's correlation analysis was used to evaluate qRT2-PCR data, as an alternative method for the grouping of PRV genes into kinetic classes. Pearson's correlation coefficient (r) was calculated as follows.



A correlation is a number between -1 and +1 that measures the degree of association between two variables [labeled here as X and Y, which are the R_Δ _values of two different genes in the same time interval (i)].  and  are the average values, n is the sample number, and S_X _and S_Y _are the standard deviances (errors) for X and Y, respectively. A positive value for the correlation implies a positive association and a negative value implies a negative or inverse association. Genes were clustered by using a complete linkage hierarchical clustering method with a centered correlation similarity metric with Cluster 3.0 software (Stanford University). To view the clustering results generated by Cluster 3.0, we used Alok Saldanha's Java TreeView.

### Gel electrophoresis

Larger DNA fragments generated by conventional PCR were run on 1% agarose/TBE gels containing ethidium bromide and visualized under UV illumination, using Marker 16 (Lambda DNA/*Eco*130I; Fermentas) to size DNA fragments. Smaller DNA fragments generated by qRT2-PCR were run in a 12% polyacrylamide gel to ensure that the amplified products had the correct size. DNA fragments were visualized with ethidium bromide staining under UV illumination. A GeneRuler™ Low Range DNA Ladder (Fermentas) was included in each run.

### Restriction endonuclease analysis

If there were doubts concerning the identity of the amplified product, restriction endonuclease analysis was performed to confirm the specificity of the qRT2-PCR products.

### Construction of recombinant plasmids

Plasmids containing PRV DNA fragments were constructed for DNA sequencing analysis, which was performed if primers designed on the basis of sequence data relating to non-Ka strains performed badly; or if the identity of amplicons generated by real-time PCR was uncertain. PRV DNAs were subcloned by using two methods: PCR amplification of the particular DNA segment; or subcloning of the desired DNA region by standard molecular cloning protocols. Amplified products were subcloned to the pGEM (Promega) vector in accordance with the manufacturer's instructions. PRV *Bam*HI fragments to be sequenced were subcloned to the pRL525 vector [67].

### DNA sequencing

Subcloned DNA fragments were subjected to DNA sequencing with the ABI Prism™ 3730xl DNA sequencer (AME Bioscience Ltd.). DNA sequences were analyzed by using the Chromas Lite 2.01 software (Technelysium Pty Ltd).

## Competing interests

The authors declare that they have no competing interests.

## Authors' contributions

DT carried out the standard and real-time PCR, the agarose and polyacrylamide gel electrophoresis, and the DNA sequencing, and participated in the evaluation of the primary data. JT took part by performing the reverse transcription reactions, purified PRV RNA, and propagated PK-15 cells. PP participated in performing the reverse transcription reactions. ZB coordinated the study, propagated viruses and isolated viral DNAs. All authors have read and approved the final manuscript.

## Supplementary Material

Additional file 1 **Validation of qRT2-PCR products by restriction endonuclease analysis**. In a few cases, the specificity of PCR products was confirmed by restriction endonuclease assay. Polyacrylamide gel electrophoresis of qRT2-PCR products, using primers ul54 (lanes 1 and 2) and us4 (lanes 3 and 4). Amplicon specificity was confirmed by restriction endonuclease analysis, using SalI (lane 2) and HpaII (lane 4) enzymes. The GeneRuler™ Low Range DNA Ladder is shown on the left. Click here for file

Additional file 2 **Grouping PRV genes according to their expression in untreated cells**. Data of R values of untreated samples. Click here for file

Additional file 3 **PRV genes ranked on the basis of the effect of PAA on gene expression**. PRV genes were classified on the basis of the inhibitory effect of PAA (R_i-PAA_) on gene expression. Early genes are separated by E/L genes from late genes. The *ie180 *gene was classified as IE gene because its expression was not inhibited by CHX treatment. ^a ^R values of PAA-treated samples at 4 h post-infection. ^b ^Relative expression ratios of PRV genes after PAA-treatment at 6 h post-infection. ^c ^R_i-PAA _values at 4 h post-infection. ^d ^PRV genes ranked on their R_i-PAA _(6 h pi) values. ^e ^R_i-PAA _values of PRV genes after 6 hours post-infection. Click here for file

Additional file 4 **PRV genes ranked on the basis of their expression profiles**. a. R values Genes were ranked on the basis of their R values at different time points. ^a ^PRV genes ranked on the basis of R values at 1 h pi. ^b ^Relative expression ratios of PRV genes at 1 h pi. ^c ^Order of R values of PRV genes at 2 h pi. ^d ^R values after 2 hours post infection. ^e ^PRV genes ranked on the basis of R values at 4 h pi. ^f ^R values at 4 h pi. b. R_Δ _values Genes were ranked on the basis of their R_Δ _values at different time intervals. ^a ^Order of R_Δ _values of PRV genes in the interval 1 h-2 h. ^b ^R_Δ _values in the interval between 1 and 2 h. ^c ^PRV genes ranked on the basis of their R_Δ _values in the infection period from 2 to 4 hours. ^d ^R_Δ _values in the interval 2 h-4 h. ^e ^PRV genes ranked on their basis of their R_Δ _values in the interval 4 h-6 h. ^f ^R_Δ _values in the infection period: 4 h-6 h. c. R_a _values Genes were ranked on the basis of their R_a _values at different time intervals. ^a ^Order of R_a _values of PRV genes in the interval 1 h-2 h. ^b ^R_a _values in the interval between 1 and 2 hours. ^c ^PRV genes ranked on the basis of their R_a _values in the infection period from 2 to 4 hours. ^d ^R_a _values in the infection period from 2 to 4 hours. ^e ^PRV genes ranked on their basis of their R_a _values in the interval 4 h-6 h. ^f ^R_a _values in the interval 4 h-6 h. Click here for file

Additional file 5 **PRV genes ranked on the basis of their R_(6h-4h)_/R_1h _ratios**. R_(6h-4h)_/R_1h _values. Click here for file

Additional file 6 **Average cycle threshold (a) and amplification values (b) with standard errors**.^a ^Average of cycle threshold (Ct) values based on almost three separated reactions. ^b ^Standard error of the mean. Click here for file
