# Supplementary figures and images for: Whole-genome analysis of pseudorabies virus gene expression by real-time quantitative RT-PCR assay
Source: BMC Genomics. 2009 Oct 23;10:491. doi: 10.1186/1471-2164-10-491 (PMC2775753; doi:10.1186/1471-2164-10-491)

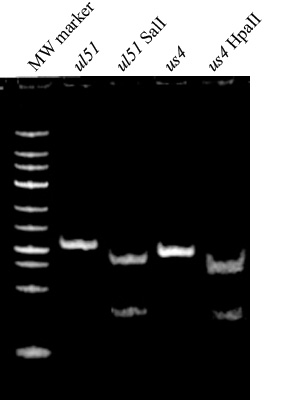

Supplement: Additional file 1 — Validation of qRT2-PCR products by restriction endonuclease analysis. In a few cases, the specificity of PCR products was confirmed by restriction endonuclease assay. Polyacrylamide gel electrophoresis of qRT2-PCR products, using primers ul54 (lanes 1 and 2) and us4 (lanes 3 and 4). Amplicon specificity was confirmed by restriction endonuclease analysis, using SalI (lane 2) and HpaII (lane 4) enzymes. The GeneRuler™ Low Range DNA Ladder is shown on the left. [file 1471-2164-10-491-S1.JPEG]
