# Supplementary material for: Whole-genome analysis of pseudorabies virus gene expression by real-time quantitative RT-PCR assay
Source: BMC Genomics. 2009 Oct 23;10:491. doi: 10.1186/1471-2164-10-491 (PMC2775753; doi:10.1186/1471-2164-10-491)
Supplement: Additional file 2 — Grouping PRV genes according to their expression in untreated cells. Data of R values of untreated samples. [file 1471-2164-10-491-S2.DOC]

**1 h pi** The *ul30* (DNA polymerase) gene reaches almost half (46.4%) of its maximal amount of transcripts by the first hour of PRV infection (Additional file 4a). *ul30* is described as an E gene in the HSV, but it has not yet been characterized in the PRV. The *ie180* gene has the second highest R_1 h_ value (38.6%) followed by the *llt1* (27.5%), *ul36* (19.5%) and *ul29* (19.1%) genes. The R_1 h_ value of *us1* is very low (0.2%), indicating that this gene is far from its peak at 1 h pi. Typical L genes can be found at the end of the list: *ul7*, *ul16*, *ul53* (each 0%), and *ul25* (0.1%). We assume that low primer efficiencies explain these zero values, and the real expression levels of these 3 genes are slightly higher (but still very low). Altogether, typical E genes can be found at the top and typical L genes at the bottom of the list containing the R_1 h_ values in decreasing sequence.

**2 h pi** Similarly as at 1 h pi, the *ul30* gene has the highest R_2 h_ value (70.5%) among the protein-encoding genes (Additional file 4a). Intriguingly, the net increase of the *ul30* transcripts is also the highest (R_(2 h-1 h)_ = 24.1%) between 1 and 2 h (Additional file 4b). The *ul30* transcripts are highly abundant, as indicated by the low Ct values in each investigation period. The above results show that the majority of DNA polymerase mRNAs are already produced in the E stage of infection. The *ul11* gene, with its second highest score (22.9%), appears to be an exception because it is an L gene in the HSV. However, PRV *ul11* behaves as an E gene, as also indicated by its relatively high R_1 h_ value and the low inhibitory effect of PAA on its expression. Furthermore, *ul11* was described as an E/L gene by the Wagner group in another publication [36]. The *us1* gene has relatively high R_2 h_ (11%) and R_(2 h-1 h)_ (10.8%) values, which is a characteristic of E genes. However, the Ct value of *us1* is relatively high, indicating a low amount of transcript in the E phase of infection. Nonetheless, the high Ct value can also be explained by low primer efficiency. The transcripts of *ul7*, *ul16* and *ul53* are still undetectable in this phase of infection. Several other L gene products do not increase significantly between 1 and 2 h pi (R_(2 h-1 h)_ ~ 0: *orf-1*, *ul3.5*, *ul10*, *ul17, ul25*, *ul26*, *ul33* and *ul44*). The IE180 mRNAs are the only PRV transcripts whose amounts significantly decline (by 27.6%) within the interval 1-2 h pi. Surprisingly, several L genes have higher R_(2 h/1 h)_ values (Additional file 4c) than any of the E genes (except *us*, which has far the highest value); this is explained by the observation that these L genes have very low R_1 h_ values, and therefore (even though their R_2 h_ values are lower than those of E genes) their ratios R_2 h_/R_1 h_ ratios give large numbers. Generally, L genes have low, whereas E genes have high R_2 h_ values; and the net increase (R_Δ_) between 1 and 2 h pi is typically higher for E than for L genes. The antisense LLT2 transcripts peak at 2 h pi, so it has higher values than any mRNAs. The LLT1 expression is also very high at 2 h pi. The two LLTs have higher R_(2 h-1 h)_ values than any of the mRNAs.

**4 h pi** The R_4 h_ values demonstrate that the E genes are close to their peaks, but the L genes are still far from them by 4 h pi (Additional file 4a). *ul53* is a typical L gene in the HSV. On the other hand, Baumeister *et al*. [40] detected the PRV homolog between 2 and 3 h pi by Northern blot hybridization, which suggests that this gene is expressed with E kinetics in the PRV. Although there is no detectable *ul53* activity in the first 2 h, its mRNA level rises to close to the maximal value by 4 h. Together with the R_i-PAA_ data (a medium level of inhibition), *ul53* appears to display E/L expression kinetics. Similarly to R_1 h_ and R_2 h_, the R_4 h_ values of the *ul11* and *ul21* genes are relatively high, indicating that, unlike HSV, these genes exhibit E expression kinetics in the PRV. The same result (E genes) is obtained for these genes by PAA analysis. The *ul38* gene is expressed in a somewhat irregular manner: while it produces a relatively high amount of transcripts in the 2 h pi (in agreement with the data obtained in Northern blot experiments by Braun et al. [46]), which is an E characteristic, its expression level is far from the maximum at 4 h, which is an L characteristic. In view of the very strong effect of PAA on *ul38*, it is classified as an L gene. The expressions of *ul36* and *ul30* also stagnate between 2 and 4 h (R _(4 h-2 h)_ ~ 0; Additional file 4b). *llt1* and *llt2* have the lowest R_4 h_ value, but these transcripts exhibit a complex pattern of gene expression, and accordingly, these values are not informative with regard to the kinetic classification. The expressions of L genes generally alter faster (high R_a_ values; Additional file 4c), than those of E genes in the 2-4 h infection period. On the basis of microarray data, Flori and colleagues [27] assumed an important role of the *ul49.5* gene in the E phase of PRV infection. We cannot confirm this result because this gene exhibits low R values until 4 h pi (1 h: 0.2%; 2 h: 3.6%; 4 h: 33.7%). However, *ul49.5* displays a very high rate of change between 1 and 2 h (R_(2 h/1 h)_ = 18, the second highest value) and between 2 and 4 h (R_(4 h/2 h)_ = 9.361), which are both L characteristics. Additionally, *ul49.5* has a comparatively low Ct value in the E phase of infection. The low R_i-PAA_, R_1 h_, R_2 h_ and R_4 h_ values of *orf-1* indicate that it is an L gene. Thus, our data reveal that the rates of change (R_a_) of L gene transcripts are higher than those of E genes in the 2-4 h infection period, which is explained by the low R values of the L genes at 2 h pi.

**6 h pi** *us3* is the only PRV gene that peaks at 4 h, and declines at 6 h pi (R_(6 h-4 h)_(%) = -16.1%; Additional file 4b). Furthermore, 4 E genes are close to their maximal value by 4 h pi (R_(6 h-4 h)_(%): 13% for *ul23*; 13.1% for *ul29*; 14.2% for *ep0* and 18.7% for *ul28*; Additional file 4b) in the examined 0-6 h infection period. The *ul16* gene displays far the highest fold change from 4 to 6 h (R_(6 h/4 h)_ = 71.865). Except for *us1* (R_(6 h/4 h)_ = 9.299), HSV genes with high R_(6 h/4 h)_ values belong in the L kinetic class. The *ul38* (R_(6/4)_ = 10.439) and *ul10* (R_(6 h/4 h)_ = 6.991) genes were earlier characterized as E genes in the PRV, which was not confirmed by our data. The *orf-1* gene has a high R_a_ value (8.528), which further confirms that this gene is expressed with L kinetics. A typical characteristic feature of L gene products is that their R_Δ_ values are low in the 0-1 h and high in the 4-6 h infection period. Thus, the ratio R_6 h-4 h)_/R_(1 h-0h)_ [= R_(6 h-4 h)_/R_1 h_] is expected to give high values for L genes and low values for E genes. Indeed, when these values were ranked in decreasing sequence, the upper part of the list contained the L genes while the E genes were located at the bottom part of the list (Additional file 5). An important exception is the *us1* gene, which has by far the highest value (683) if we neglect the *ul7*, *ul16* and *ul53* genes, which have R_1 h_=0 values in the denominator therefore giving infinite R_(6 h-4 h)_/R_1 h_ values. The following genes have the highest ratios (R_(6 h-4 h)_)/R_1 h_: *ul25* (468), *ul49.5* (409.5), *orf-1* (406.5), *ul44* (382.5) and *ul17* (376.667). The lowest values were as follows: *us3* (-1.095), *ul29* (0.686), *ie180* (1.462), *ul30* (1.515), *ep0* (1.868) and *ul23* (2.549). Thus, according to our data, in the 4-6 h infection period, both the net increase (R_Δ_) and the rate of change (R_a_) of the L gene transcripts are higher than those of the E genes. Comparison of the ranking of the genes on the basis of their R_6 h_–R_4 h_/R_1 h_ and R_i-PAA_ values indicates a significant similarity, which means that viral genes can be classified into the same groups by analyzing the gene expression without drug treatment.
