# Supplementary material for: Whole-genome analysis of pseudorabies virus gene expression by real-time quantitative RT-PCR assay
Source: BMC Genomics. 2009 Oct 23;10:491. doi: 10.1186/1471-2164-10-491 (PMC2775753; doi:10.1186/1471-2164-10-491)
Supplement: Additional file 3 — PRV genes ranked on the basis of the effect of PAA on gene expression. PRV genes were classified on the basis of the inhibitory effect of PAA (Ri-PAA) on gene expression. Early genes are separated by E/L genes from late genes. The ie180 gene was classified as IE gene because its expression was not inhibited by CHX treatment. a R values of PAA-treated samples at 4 h post-infection. b Relative expression ratios of PRV genes after PAA-treatment at 6 h post-infection. c Ri-PAA values at 4 h post-infection. d PRV genes ranked on their Ri-PAA (6 h pi) values. e Ri-PAA values of PRV genes after 6 hours post-infection. [file 1471-2164-10-491-S3.PDF]

Additional File 3. PRV genes ranked on the basis of PAA effect on gene expression

4h PAA[a]	6hPAA[b]	4h PAA/untreated[c]	gene[d]	6h PAA/untreated[e]
0.347	0.374	38.556	***llt2***	2.904
1.446	1.198	2.123	***ul30***	0.876
0.223	0.492	0.437	***ul23***	0.775
0.622	0.796	0.514	***us3***	0.762
0.140	0.972	0.187	***ul11***	0.759
0.559	0.818	1.090	***ul8***	0.755
0.269	0.757	0.378	***ul21***	0.680
0.383	0.697	0.364	***ul29***	0.593
0.221	0.481	0.328	***ul28***	0.564
0.019	0.728	0.063	***ul13***	0.553
0.373	0.563	0.621	***ul9***	0.510
0.376	0.532	0.619	***ul54***	0.505
0.319	0.478	0.539	***ul40***	0.500
0.322	0.399	0.478	***ep0***	0.493
0.263	0.479	0.394	***ul50***	0.489
0.334	0.443	0.612	***ul12***	0.426
0.274	0.486	1.038	***us6***	0.390
1.053	0.784	1.127	***ul52***	0.389
0.171	0.521	0.510	***ul7***	0.373
0.322	0.455	0.803	***ul15***	0.366
0.207	0.361	0.555	***ul39***	0.328
0.203	0.359	0.375	***ul14***	0.325
0.297	0.357	0.627	***ul4***	0.316
0.325	0.335	0.450	***ul43***	0.312
0.191	0.461	0.166	***ul53***	0.304
0.296	0.280	0.578	***us4***	0.282
0.123	0.294	0.335	***us8***	0.255
0.100	0.227	0.508	***us2***	0.237
0.149	0.228	0.578	***us7***	0.227
0.128	0.227	0.261	***ie180***	0.218
0.172	0.212	0.415	***ul46***	0.215
0.086	0.363	0.223	***ul20***	0.205
0.095	0.203	0.403	***us9***	0.202
0.068	0.195	0.191	***ul32***	0.194
0.106	0.208	0.218	***ul42***	0.194
0.134	0.206	0.472	***ul3***	0.187
0.171	0.184	0.594	***ul36***	0.183
0.082	0.187	0.152	***ul34***	0.175
0.039	0.155	0.364	***orf-1***	0.171
0.091	0.181	0.337	***ul49***	0.168
0.175	0.212	0.364	***ul33***	0.162
0.322	0.181	0.668	***ul41***	0.160
0.094	0.180	0.367	***ul35***	0.159
0.122	0.173	0.351	***ul18***	0.148
0.078	0.187	0.255	***ul19***	0.147
0.186	0.193	0.579	***ul27***	0.144
0.129	0.155	0.401	***ul48***	0.141
0.053	0.159	0.159	***ul49.5***	0.140
0.174	0.129	0.989	***ul2***	0.129
0.200	0.162	0.545	***ul5***	0.126
0.042	0.142	0.627	***ul31***	0.125
0.074	0.145	0.303	***ul22***	0.124
0.079	0.138	0.271	***ul6***	0.112
0.038	0.074	0.167	***ul25***	0.108
0.022	0.133	0.088	***ul17***	0.098
0.026	0.094	0.138	***ul26***	0.083
0.057	0.088	0.377	***ul24***	0.075
0.087	0.085	0.370	***ul47***	0.072
0.015	0.093	0.034	***ul3.5***	0.070
0.054	0.101	0.260	***ul10***	0.070
0.036	0.063	0.221	***ul37***	0.061
0.032	0.050	0.128	***ul51***	0.052
0.008	0.040	0.051	***ul44***	0.044
0.078	0.066	0.476	***us1***	0.044
0.009	0.044	0.093	***ul38***	0.044
0.066	0.076	0.247	***ul1***	0.042
0.075	0.001	3.000	***llt1***	0.005
0.000	0.000	0.000	***ul16***	0.000
