# Supplementary material for: Whole-genome analysis of pseudorabies virus gene expression by real-time quantitative RT-PCR assay
Source: BMC Genomics. 2009 Oct 23;10:491. doi: 10.1186/1471-2164-10-491 (PMC2775753; doi:10.1186/1471-2164-10-491)
Supplement: Additional file 4 — PRV genes ranked on the basis of their expression profiles. a. R values Genes were ranked on the basis of their R values at different time points. a PRV genes ranked on the basis of R values at 1 h pi. b Relative expression ratios of PRV genes at 1 h pi. c Order of R values of PRV genes at 2 h pi. d R values after 2 hours post infection. e PRV genes ranked on the basis of R values at 4 h pi. f R values at 4 h pi. b. RΔ values Genes were ranked on the basis of their RΔ values at different time intervals. a Order of RΔ values of PRV genes in the interval 1 h-2 h. b RΔ values in the interval between 1 and 2 h. c PRV genes ranked on the basis of their RΔ values in the infection period from 2 to 4 hours. d RΔ values in the interval 2 h-4 h. e PRV genes ranked on their basis of their RΔ values in the interval 4 h-6 h. f RΔ values in the infection period: 4 h-6 h. c. Ra values Genes were ranked on the basis of their Ra values at different time intervals. a Order of Ra values of PRV genes in the interval 1 h-2 h. b Ra values in the interval between 1 and 2 hours. c PRV genes ranked on the basis of their Ra values in the infection period from 2 to 4 hours. d Ra values in the infection period from 2 to 4 hours. e PRV genes ranked on their basis of their Ra values in the interval 4 h-6 h. f Ra values in the interval 4 h-6 h. [file 1471-2164-10-491-S4.PDF]

Additional File 4a. PRV genes ranked on the basis of their R values

gene[a]	1h[b]	gene[c]	2h[d]	gene[e]	4h[f]
ul30	0.458	*ul30*	0.713	*us3*	1.209
ie180	0.380	*llt2*	0.484	*ul53*	1.152
ul36	0.192	*ul29*	0.393	*ul29*	1.051
ul29	0.188	*us3*	0.368	*ul52*	0.934
us3	0.145	*ul36*	0.328	*ul11*	0.749
ul8	0.105	*ul43*	0.266	*ul43*	0.723
ep0	0.075	*ul11*	0.263	*ul21*	0.712
ul9	0.070	*ul9*	0.250	*ul30*	0.681
ul54	0.062	*ul8*	0.228	*ep0*	0.674
ul28	0.056	*ul49*	0.201	*ul28*	0.673
ul21	0.050	*ul50*	0.179	*ul50*	0.668
ul23	0.050	*llt1*	0.177	*ul54*	0.607
ul33	0.050	*ul34*	0.176	*ul9*	0.601
ul40	0.047	*ul54*	0.157	*ul40*	0.592
ul14	0.045	*ep0*	0.148	*ul12*	0.546
ul50	0.045	*ul38*	0.136	*ul14*	0.541
ul43	0.043	*ul14*	0.129	*ul34*	0.540
ul49	0.039	*ul4*	0.129	*ul8*	0.513
ul3.5	0.036	*ul40*	0.123	*us4*	0.512
ul4	0.036	*ul48*	0.116	*ul23*	0.510
us6	0.034	*us1*	0.111	*ie180*	0.491
llt1	0.033	*ie180*	0.110	*ul42*	0.487
ul2	0.032	*ul39*	0.105	*ul41*	0.482
ul11	0.031	*ul12*	0.102	*ul33*	0.481
ul38	0.031	*ul52*	0.099	*ul4*	0.474
llt2	0.029	*ul13*	0.094	*ul3.5*	0.443
ul12	0.029	*ul23*	0.093	*ul46*	0.414
ul48	0.029	*ul21*	0.091	*ul15*	0.401
ul46	0.028	*ul5*	0.088	*ul20*	0.385
ul39	0.025	*ul28*	0.078	*ul39*	0.373
ul52	0.024	*ul46*	0.074	*ul5*	0.367
ul13	0.023	*ul41*	0.072	*us8*	0.367
ul5	0.023	*us8*	0.068	*ul32*	0.356
us4	0.022	*us2*	0.057	*ul18*	0.348
us8	0.022	*us6*	0.057	*ul7*	0.335
ul1	0.021	*ul42*	0.056	*ul49.5*	0.333
ul20	0.016	*ul15*	0.053	*ul48*	0.322
us7	0.016	*us9*	0.052	*ul27*	0.321
us9	0.016	*ul3*	0.049	*ul19*	0.306
ul35	0.014	*us4*	0.048	*ul13*	0.300
ul34	0.013	*ul33*	0.045	*ul6*	0.291
ul10	0.012	*ul2*	0.042	*ul36*	0.288
ul27	0.012	*ul3.5*	0.039	*ul3*	0.284
ul26	0.011	*ul35*	0.038	*ul49*	0.270
ul3	0.011	*ul32*	0.037	*ul1*	0.267
ul42	0.011	*ul6*	0.037	*us6*	0.264
ul22	0.009	*ul19*	0.036	*us7*	0.258
ul24	0.009	*ul49.5*	0.036	*ul35*	0.256
ul41	0.009	*us7*	0.035	*ul17*	0.250
ul15	0.007	*ul20*	0.034	*ul51*	0.250
ul19	0.007	*ul31*	0.032	*ul22*	0.244
ul31	0.007	*ul27*	0.028	*us9*	0.236
ul18	0.006	*ul1*	0.027	*ul47*	0.235
ul32	0.006	*ul18*	0.027	*ul25*	0.227
ul51	0.005	*ul22*	0.026	*ul10*	0.208
us2	0.005	*ul37*	0.026	*us2*	0.197
ul37	0.004	*ul47*	0.016	*ul26*	0.189
ul47	0.004	*ul26*	0.012	*ul2*	0.176
ul17	0.003	*ul24*	0.011	*us1*	0.164
ul6	0.003	*ul10*	0.010	*ul37*	0.163
orf-1	0.002	*ul51*	0.010	*ul44*	0.158
ul44	0.002	*ul25*	0.004	*ul24*	0.151
ul49.5	0.002	*ul17*	0.003	*orf-1*	0.107
us1	0.002	*orf-1*	0.002	*ul38*	0.097
ul25	0.001	*ul44*	0.002	*ul31*	0.067
ul16	0.000	*ul16*	0.000	*ul16*	0.027
ul53	0.000	*ul53*	0.000	*llt1*	0.025
ul7	0.000	*ul7*	0.000	*llt2*	0.009

Additional File 4b. PRV genes ranked on the basis of their R_{Δ} values

gene[a]	2h-1h[b]	gene[c]	4h-2h[d]	gene[e]	6h-4h[f]
llt2	0.455	***ul53***	1.152	***ul16***	1.880
ul30	0.255	***us3***	0.841	***ul1***	1.528
ul11	0.232	***ul52***	0.835	***ul20***	1.384
ul43	0.223	***ul29***	0.658	***us1***	1.342
us3	0.223	***ul21***	0.621	***ul10***	1.242
ul29	0.205	***ul28***	0.595	***ul17***	1.110
ul9	0.180	***ep0***	0.526	***ul52***	1.079
ul34	0.163	***ul50***	0.489	***ul31***	1.072
ul49	0.162	***ul11***	0.486	***ul7***	1.063
llt1	0.144	***ul40***	0.469	***ul24***	1.018
ul36	0.136	***us4***	0.464	***ul27***	1.017
ul50	0.134	***ul43***	0.457	***ul13***	1.016
ul8	0.123	***ul54***	0.450	***us6***	0.983
us1	0.109	***ul12***	0.444	***ul19***	0.964
ul38	0.105	***ul33***	0.436	***ul47***	0.948
ul54	0.095	***ul42***	0.431	***ul6***	0.947
ul4	0.093	***ul23***	0.417	***ul26***	0.939
ul48	0.087	***ul14***	0.412	***ul22***	0.930
ul14	0.084	***ul41***	0.410	***ul5***	0.918
ul39	0.080	***ul3.5***	0.404	***ul38***	0.909
ul40	0.076	***ie180***	0.381	***ul3.5***	0.884
ul52	0.075	***ul34***	0.364	***ul35***	0.879
ep0	0.073	***ul9***	0.351	***ul37***	0.865
ul12	0.073	***ul20***	0.351	***ul15***	0.841
ul13	0.071	***ul15***	0.348	***ul33***	0.827
ul5	0.065	***ul4***	0.345	***ul2***	0.827
ul41	0.063	***ul46***	0.340	***ul3***	0.820
us2	0.052	***ul7***	0.335	***ul18***	0.819
ul15	0.046	***ul18***	0.321	***ul49***	0.804
ul46	0.046	***ul32***	0.319	***ul49.5***	0.803
us8	0.046	***us8***	0.299	***orf-1***	0.798
ul42	0.045	***ul49.5***	0.297	***us8***	0.788
ul23	0.043	***ul27***	0.293	***ul48***	0.781
ul21	0.041	***ul8***	0.285	***us9***	0.770
ul3	0.038	***ul5***	0.279	***us2***	0.760
us9	0.036	***ul19***	0.270	***ul44***	0.751
ul49.5	0.034	***ul39***	0.268	***us7***	0.749
ul6	0.034	***ul6***	0.254	***ul39***	0.729
ul32	0.031	***ul17***	0.247	***ul36***	0.718
ul19	0.029	***ul1***	0.240	***ul51***	0.716
us4	0.026	***ul51***	0.240	***ul30***	0.687
ul31	0.025	***ul3***	0.235	***ul4***	0.657
ul35	0.024	***ul25***	0.223	***ul41***	0.651
us6	0.023	***us7***	0.223	***ul32***	0.649
ul28	0.022	***ul47***	0.219	***ul42***	0.586
ul37	0.022	***ul22***	0.218	***ul46***	0.574
ul18	0.021	***ul35***	0.218	***ul8***	0.570
us7	0.019	***us6***	0.207	***ul14***	0.565
ul20	0.018	***ul48***	0.206	***ie180***	0.551
ul22	0.017	***ul13***	0.206	***ul11***	0.531
ul27	0.016	***ul10***	0.198	***ul34***	0.526
ul47	0.012	***us9***	0.184	***ul9***	0.502
ul2	0.010	***ul26***	0.177	***ul12***	0.493
ul1	0.006	***ul44***	0.156	***us4***	0.481
ul51	0.005	***ul24***	0.140	***ul25***	0.459
ul25	0.003	***us2***	0.140	***ul54***	0.447
ul3.5	0.003	***ul37***	0.137	***ul21***	0.401
ul24	0.002	***ul2***	0.134	***ul53***	0.367
ul26	0.001	***orf-1***	0.105	***ul40***	0.364
ul44	0.000	***ul49***	0.069	***ul43***	0.351
ul53	0.000	***us1***	0.053	***ul50***	0.312
orf-1	0,000	***ul31***	0.035	***ul28***	0.180
ul16	0,000	***ul16***	0.027	***llt1***	0.177
ul17	0,000	***ul30***	-0.032	***ep0***	0.136
ul7	0,000	***ul38***	-0.039	***ul23***	0.125
ul10	-0.002	***ul36***	-0.040	***ul29***	0.124
ul33	-0.005	***llt1***	-0.152	***llt2***	0.120
ie180	-0.270	***llt2***	-0.475	***us3***	-0.164

Additional File 4c. PRV genes ranked on the basis of their R_a values

gene[a]	2h/1h[b]	gene[c]	4h/2h[d]	gene[e]	6h/4h[f]
us1	55.500	***ul17***	83.333	***ul16***	70.630
ul49.5	18.000	***ul44***	79.000	***ul31***	17.004
llt2	16.690	***ul25***	56.750	***llt2***	14.308
ul34	13.538	***orf-1***	53.500	***ul38***	10.367
ul6	12.333	***ul51***	25.000	***us1***	9.183
us2	11.400	***ul10***	20.800	***orf-1***	8.461
ul11	8.484	***ul26***	15.750	***llt1***	8.061
ul41	8.000	***ul47***	14.688	***ul24***	7.740
ul15	7.571	***ul24***	13.727	***ul10***	6.971
ul37	6.500	***ul18***	12.889	***ul1***	6.726
ul32	6.177	***ul27***	11.464	***ul37***	6.308
ul43	6.176	***ul3.5***	11.359	***ul26***	5.971
llt1	5.364	***ul20***	11.324	***ul44***	5.755
ul19	5.154	***ul33***	10.689	***ul2***	5.697
ul42	5.143	***us4***	10.667	***ul17***	5.438
ul49	5.091	***ul1***	9.889	***ul47***	5.032
ul31	4.571	***ul32***	9.622	***us2***	4.860
ul18	4.500	***ul52***	9.434	***ul22***	4.810
ul3	4.455	***ul22***	9.385	***us6***	4.725
ul38	4.387	***ul49.5***	9.250	***ul20***	4.596
ul39	4.200	***ul42***	8.696	***ul35***	4.435
ul52	4.125	***ul28***	8.628	***ul13***	4.387
ul13	4.087	***ul19***	8.500	***us9***	4.261
ul25	4.000	***ul6***	7.865	***ul6***	4.253
ul47	4.000	***ul21***	7.824	***ul7***	4.173
ul48	4.000	***ul15***	7.566	***ul27***	4.168
ul50	3.978	***us7***	7.371	***ul19***	4.150
ul5	3.826	***ul41***	6.701	***ul49***	3.979
ul4	3.583	***ul35***	6.990	***us7***	3.902
ul9	3.571	***ul37***	6.269	***ul3***	3.887
ul12	3.517	***ul3***	5.796	***ul51***	3.865
us9	3.250	***ul46***	5.595	***ul5***	3.501
us8	3.091	***ul23***	5.484	***ul36***	3.492
ul22	2.889	***us8***	5.397	***ul48***	3.425
ul14	2.867	***ul12***	5.353	***ul49.5***	3.412
ul35	2.714	***ul40***	4.813	***ul18***	3.353
ul46	2.643	***us6***	4.632	***us8***	3.147
ul40	2.617	***us9***	4.554	***ul15***	3.096
us3	2.538	***ep0***	4.538	***ul25***	3.023
ul54	2.532	***ie180***	4.464	***ul3.5***	2.996
ul27	2.333	***ul14***	4.194	***ul39***	2.954
us7	2.188	***ul2***	4.190	***ul32***	2.822
us4	2.182	***ul5***	4.170	***ul33***	2.720
ul8	2.171	***ul54***	3.866	***ul46***	2.387
ul20	2.125	***ul50***	3.732	***ul4***	2.386
ul29	2.090	***ul4***	3.674	***ul41***	2.351
ul51	2.000	***ul39***	3.552	***ul42***	2.204
ep0	1.973	***us2***	3.456	***ul52***	2.155
ul23	1.860	***us3***	3.285	***ie180***	2.122
ul21	1.820	***ul13***	3.191	***ul8***	2.112
ul36	1.708	***ul34***	3.068	***ul14***	2.044
us6	1.676	***ul11***	2.848	***ul30***	2.009
ul30	1.557	***ul48***	2.776	***ul34***	1.973
ul28	1.393	***ul43***	2.718	***us4***	1.939
ul2	1.313	***ul29***	2.674	***ul12***	1.903
ul1	1.286	***ul9***	2.404	***ul9***	1.835
ul24	1.222	***ul8***	2.250	***ul54***	1.736
ul26	1.091	***ul31***	2.094	***ul11***	1.709
ul3.5	1.083	***us1***	1.477	***ul40***	1.616
orf-1	1.000	***ul49***	1.343	***ul21***	1.563
ul17	1.000	***ul30***	0.955	***ul43***	1.486
ul44	1.000	***ul36***	0.878	***ul50***	1.467
ul33	0.900	***ul38***	0.713	***ul53***	1.318
ul10	0.833	***llt1***	0.141	***ul28***	1.268
ie180	0.289	***llt2***	0.019	***ul23***	1.245
ul16	0.000	***ul16***	0.000	***ep0***	1.202
ul53	0.000	***ul53***	0.000	***ul29***	1.118
ul7	0.000	***ul7***	0.000	***us3***	0.864
