# Supplementary material for: Whole-genome analysis of pseudorabies virus gene expression by real-time quantitative RT-PCR assay
Source: BMC Genomics. 2009 Oct 23;10:491. doi: 10.1186/1471-2164-10-491 (PMC2775753; doi:10.1186/1471-2164-10-491)
Supplement: Additional file 5 — PRV genes ranked on the basis of their R(6h-4h)/R1h ratios. R(6h-4h)/R1h values. [file 1471-2164-10-491-S5.PDF]

Additional file 5. PRV genes ranked on the basis of their $R_{(6\ h-4\ h)}/R_{1\ h}$ ratios

**genee**	**$R_{(6\ h-4\ h)}$ / R_{1h}**
ul16	∞
ul53	∞
ul7	∞
us1	670.978
ul25	459.134
ul49.5	401.674
orf-1	399.172
ul44	375.638
ul17	369.830
ul6	315.532
ul47	236.887
ul37	216.305
ul31	153.185
us2	152.088
ul51	143.258
ul19	137.719
ul18	136.470
ul15	120.076
ul24	113.087
ul32	108.104
ul10	103.494
ul22	103.300
ul20	86.525
ul26	85.408
ul27	84.739
ul3	74.537
ul1	72.807
ul41	72.378
ul35	62.812
ul42	53.312
us9	48.101
us7	46.787
ul52	44.966
ul13	44.184
ul34	40.429
ul5	39.904
us8	35.819
ul38	29.310
ul39	29.158
us6	28.924
ul48	26.928
ul2	25.833
ul3.5	24.557
us4	21.856
ul49	20.626
ul46	20.497
ul4	18.262
ul11	17.125
ul12	17.001
ul33	16.547
ul14	12.553
ul43	8.173
ul21	8.015
ul40	7.754
ul54	7.206
ul9	7.170
ul50	6.934
ul8	5.431
llt1	5.349
llt2	4.130
ul36	3.738
ul28	3.219
ul23	2.500
ep0	1.813
ul30	1.501
ie180	1.450
ul29	0.658
us3	-1.132
