# Supplementary material for: Whole-genome analysis of pseudorabies virus gene expression by real-time quantitative RT-PCR assay
Source: BMC Genomics. 2009 Oct 23;10:491. doi: 10.1186/1471-2164-10-491 (PMC2775753; doi:10.1186/1471-2164-10-491)
Supplement: Additional file 6 — Average cycle threshold (a) and amplification values (b) with standard errors.a Average of cycle threshold (Ct) values based on almost three separated reactions. b Standard error of the mean. [file 1471-2164-10-491-S6.DOC]

|  | ***orf-1*** | |  | ***ul54*** | |  | ***ul53*** | |  | ***ul52*** | |  | ***ul51*** | |  | ***ul50*** | |
| --- | --- | --- | --- | --- | --- | --- | --- | --- | --- | --- | --- | --- | --- | --- | --- | --- | --- |
| **Time** | **Ct ^a^** | **SE ^b^** |  | **Ct** | **SE** |  | **Ct** | **SE** |  | **Ct** | **SE** |  | **Ct** | **SE** |  | **Ct** | **SE** |
| **1h** | 28.53 | 0.16 |  | 26.33 | 0.58 |  | 0.00 | 0.00 |  | 28.43 | 0.13 |  | 28.55 | 0.11 |  | 26.20 | 0.56 |
| **2h** | 28.48 | 0.06 |  | 25.25 | 1.21 |  | 0.00 | 0.00 |  | 27.67 | 0.12 |  | 28.45 | 0.10 |  | 23.95 | 0.25 |
| **4h** | 25.98 | 0.80 |  | 21.95 | 0.51 |  | 21.20 | 0.14 |  | 25.45 | 0.56 |  | 26.68 | 0.45 |  | 22.05 | 0.74 |
| **4h PAA** | 27.45 | 0.50 |  | 23.28 | 0.18 |  | 23.10 | 0.10 |  | 27.08 | 0.35 |  | 28.13 | 0.03 |  | 24.73 | 1.26 |
| **6h** | 21.95 | 0.21 |  | 21.23 | 0.24 |  | 20.55 | 0.38 |  | 25.00 | 0.94 |  | 24.98 | 0.22 |  | 21.03 | 0.31 |
| **6h PAA** | 25.95 | 0.36 |  | 22.68 | 0.51 |  | 22.88 | 0.31 |  | 25.77 | 0.16 |  | 27.90 | 0.17 |  | 22.40 | 0.54 |
|  |  |  |  |  |  |  |  |  |  |  |  |  |  |  |  |  |  |
|  | ***ul49.5*** | |  | ***ul49*** | |  | ***ul48*** | |  | ***ul47*** | |  | ***ul46*** | |  | ***ul27*** | |
|  | **Ct** | **SE** |  | **Ct** | **SE** |  | **Ct** | **SE** |  | **Ct** | **SE** |  | **Ct** | **SE** |  | **Ct** | **SE** |
| **1h** | 28.68 | 0.02 |  | 24.77 | 0.19 |  | 27.05 | 0.35 |  | 28.70 | 0.00 |  | 25.50 | 0.52 |  | 27.45 | 0.57 |
| **2h** | 28.30 | 0.06 |  | 23.30 | 0.16 |  | 25.75 | 0.28 |  | 28.30 | 0.10 |  | 23.40 | 0.29 |  | 26.63 | 0.76 |
| **4h** | 27.33 | 0.50 |  | 20.77 | 0.37 |  | 23.43 | 0.46 |  | 27.58 | 0.14 |  | 21.10 | 0.81 |  | 22.48 | 0.84 |
| **4h PAA** | 28.15 | 0.03 |  | 23.00 | 0.48 |  | 25.48 | 0.19 |  | 28.18 | 0.10 |  | 22.65 | 0.32 |  | 23.83 | 0.40 |
| **6h** | 26.15 | 0.27 |  | 18.75 | 0.18 |  | 21.10 | 0.26 |  | 26.70 | 0.60 |  | 19.38 | 0.25 |  | 21.10 | 1.21 |
| **6h PAA** | 27.80 | 0.23 |  | 22.00 | 0.37 |  | 24.73 | 0.30 |  | 28.15 | 0.09 |  | 21.70 | 0.51 |  | 24.00 | 1.07 |
|  |  |  |  |  |  |  |  |  |  |  |  |  |  |  |  |  |  |
|  | ***ul28*** | |  | ***ul29*** | |  | ***ul30*** | |  | ***ul31*** | |  | ***ul32*** | |  | ***ul33*** | |
|  | **Ct** | **SE** |  | **Ct** | **SE** |  | **Ct** | **SE** |  | **Ct** | **SE** |  | **Ct** | **SE** |  | **Ct** | **SE** |
| **1h** | 32.40 | 0.57 |  | 23.70 | 0.34 |  | 25.98 | 0.50 |  | 26.65 | 0.46 |  | 28.20 | 0.11 |  | 26.28 | 0.76 |
| **2h** | 32.33 | 0.25 |  | 21.70 | 0.21 |  | 23.87 | 0.30 |  | 25.30 | 0.52 |  | 27.45 | 0.03 |  | 26.35 | 0.42 |
| **4h** | 29.77 | 1.40 |  | 20.03 | 0.91 |  | 23.83 | 1.46 |  | 22.17 | 1.11 |  | 23.78 | 0.10 |  | 22.40 | 0.74 |
| **4h PAA** | 30.78 | 0.40 |  | 21.80 | 0.23 |  | 24.05 | 0.45 |  | 25.20 | 0.74 |  | 26.93 | 0.31 |  | 24.90 | 0.62 |
| **6h** | 28.08 | 0.96 |  | 19.48 | 0.20 |  | 24.50 | 1.66 |  | 18.93 | 0.36 |  | 22.25 | 0.34 |  | 20.10 | 0.11 |
| **6h PAA** | 29.70 | 1.23 |  | 20.90 | 0.40 |  | 22.65 | 0.58 |  | 22.43 | 0.14 |  | 25.60 | 0.32 |  | 24.20 | 0.32 |
|  |  |  |  |  |  |  |  |  |  |  |  |  |  |  |  |  |  |
|  | ***ul34*** | |  | ***ul35*** | |  | ***ul36*** | |  | ***ul37*** | |  | ***ul38*** | |  | ***ul39*** | |
|  | **Ct** | **SE** |  | **Ct** | **SE** |  | **Ct** | **SE** |  | **Ct** | **SE** |  | **Ct** | **SE** |  | **Ct** | **SE** |
| **1h** | 25.93 | 0.59 |  | 25.18 | 0.60 |  | 23.68 | 0.61 |  | 28.43 | 0.17 |  | 28.70 | 0.07 |  | 27.25 | 0.42 |
| **2h** | 23.68 | 0.38 |  | 23.18 | 0.26 |  | 23.00 | 0.41 |  | 27.13 | 0.31 |  | 28.50 | 0.04 |  | 25.13 | 0.26 |
| **4h** | 19.98 | 0.26 |  | 19.63 | 0.26 |  | 22.93 | 0.21 |  | 24.00 | 0.79 |  | 27.10 | 0.83 |  | 23.25 | 0.90 |
| **4h PAA** | 22.97 | 0.52 |  | 22.05 | 0.65 |  | 24.20 | 0.65 |  | 26.95 | 0.32 |  | 28.57 | 0.06 |  | 24.68 | 0.21 |
| **6h** | 18.58 | 0.36 |  | 17.45 | 0.36 |  | 20.85 | 0.35 |  | 20.85 | 0.51 |  | 25.55 | 0.26 |  | 21.18 | 0.31 |
| **6h PAA** | 21.95 | 0.64 |  | 21.05 | 0.75 |  | 23.95 | 0.42 |  | 25.88 | 0.56 |  | 28.28 | 0.13 |  | 23.18 | 0.66 |
|  |  |  |  |  |  |  |  |  |  |  |  |  |  |  |  |  |  |
|  | ***ul40*** | |  | ***ul41*** | |  | ***ul42*** | |  | ***ul43*** | |  | ***ul44*** | |  | ***ul26*** | |
|  | **Ct** | **SE** |  | **Ct** | **SE** |  | **Ct** | **SE** |  | **Ct** | **SE** |  | **Ct** | **SE** |  | **Ct** | **SE** |
| **1h** | 25.33 | 0.57 |  | 25.33 | 0.20 |  | 27.50 | 0.30 |  | 27.88 | 0.29 |  | 28.27 | 0.08 |  | 28.67 | 0.24 |
| **2h** | 23.63 | 0.55 |  | 24.98 | 0.19 |  | 25.48 | 0.26 |  | 26.48 | 0.28 |  | 28.30 | 0.15 |  | 28.40 | 0.04 |
| **4h** | 22.50 | 2.09 |  | 22.75 | 1.21 |  | 21.43 | 0.75 |  | 23.98 | 0.59 |  | 25.03 | 0.63 |  | 24.13 | 0.98 |
| **4h PAA** | 22.60 | 0.25 |  | 23.50 | 0.23 |  | 24.10 | 0.28 |  | 25.93 | 0.51 |  | 27.90 | 0.15 |  | 27.80 | 0.27 |
| **6h** | 19.50 | 0.15 |  | 20.73 | 0.33 |  | 19.98 | 0.30 |  | 21.85 | 0.52 |  | 23.75 | 1.26 |  | 21.18 | 0.36 |
| **6h PAA** | 22.35 | 0.68 |  | 23.10 | 0.53 |  | 23.33 | 0.90 |  | 26.35 | 0.92 |  | 26.97 | 0.08 |  | 26.35 | 0.38 |
|  |  |  |  |  |  |  |  |  |  |  |  |  |  |  |  |  |  |
|  | ***ul25*** | |  | ***ul24*** | |  | ***ul23*** | |  | ***ul22*** | |  | ***ul21*** | |  | ***ul20*** | |
|  | **Ct** | **SE** |  | **Ct** | **SE** |  | **Ct** | **SE** |  | **Ct** | **SE** |  | **Ct** | **SE** |  | **Ct** | **SE** |
| **1h** | 28.68 | 0.08 |  | 28.08 | 0.28 |  | 28.00 | 0.27 |  | 27.30 | 0.17 |  | 26.93 | 0.27 |  | 31.13 | 0.84 |
| **2h** | 28.18 | 0.05 |  | 27.95 | 0.17 |  | 27.38 | 0.36 |  | 26.55 | 0.39 |  | 25.68 | 0.24 |  | 29.00 | 0.14 |
| **4h** | 24.63 | 1.13 |  | 24.45 | 0.47 |  | 24.45 | 0.71 |  | 22.58 | 1.05 |  | 22.88 | 1.15 |  | 26.13 | 0.57 |
| **4h PAA** | 26.90 | 0.12 |  | 26.90 | 0.16 |  | 26.78 | 0.48 |  | 25.05 | 0.61 |  | 23.93 | 0.31 |  | 29.88 | 1.15 |
| **6h** | 21.08 | 0.71 |  | 20.98 | 0.21 |  | 24.80 | 1.59 |  | 20.25 | 0.64 |  | 21.08 | 0.32 |  | 24.40 | 0.17 |
| **6h PAA** | 25.60 | 0.45 |  | 25.35 | 0.21 |  | 24.90 | 0.72 |  | 24.03 | 0.92 |  | 22.80 | 0.40 |  | 26.68 | 0.25 |

|  | ***ul19*** | |  | ***ul18*** | |  | ***ul17*** | |  | ***ul16*** | |  | ***ul15*** | |  | ***ul14*** | |
| --- | --- | --- | --- | --- | --- | --- | --- | --- | --- | --- | --- | --- | --- | --- | --- | --- | --- |
|  | **Ct** | **SE** |  | **Ct** | **SE** |  | **Ct** | **SE** |  | **Ct** | **SE** |  | **Ct** | **SE** |  | **Ct** | **SE** |
| 1h | 27.73 | 0.28 |  | 28.18 | 0.28 |  | 28.80 | 0.05 |  | 0.00 | 0.00 |  | 28.35 | 0.13 |  | 26.83 | 0.38 |
| 2h | 25.75 | 0.49 |  | 27.43 | 0.14 |  | 28.73 | 0.03 |  | 0.000 | 0.00 |  | 27.03 | 0.27 |  | 25.45 | 0.36 |
| 4h | 22.03 | 0.83 |  | 23.05 | 0.27 |  | 26.63 | 0.60 |  | 28.25 | 0.04 |  | 24.10 | 0.90 |  | 22.08 | 0.51 |
| 4h PAA | 25.03 | 0.63 |  | 24.48 | 0.39 |  | 28.28 | 0.05 |  | 29.50 | 0.01 |  | 25.15 | 0.28 |  | 25.13 | 0.40 |
| 6h | 19.30 | 0.32 |  | 20.28 | 0.61 |  | 23.85 | 0.37 |  | 27.15 | 0.31 |  | 22.63 | 0.36 |  | 21.03 | 0.27 |
| 6h PAA | 24.25 | 0.59 |  | 23.55 | 0.38 |  | 27.35 | 0.28 |  | 0.000 | 0.00 |  | 24.50 | 0.57 |  | 22.33 | 0.94 |
|  |  |  |  |  |  |  |  |  |  |  |  |  |  |  |  |  |  |
|  | ***ul13*** | |  | ***ul12*** | |  | ***ul11*** | |  | ***ul10*** | |  | ***ul9*** | |  | ***ul8*** | |
|  | **Ct** | **SE** |  | **Ct** | **SE** |  | **Ct** | **SE** |  | **Ct** | **SE** |  | **Ct** | **SE** |  | **Ct** | **SE** |
| 1h | 26.45 | 0.77 |  | 27.20 | 0.4 |  | 27.2 | 0.6 |  | 29.03 | 0.80 |  | 27.60 | 0.26 |  | 25.00 | 0.40 |
| 2h | 23.55 | 0.91 |  | 25.15 | 0.2 |  | 26.33 | 0.2 |  | 28.35 | 0.14 |  | 26.45 | 0.13 |  | 24.15 | 0.51 |
| 4h | 21.90 | 0.56 |  | 22.53 | 0.6 |  | 22.88 | 0.9 |  | 23.65 | 0.86 |  | 24.60 | 0.58 |  | 23.85 | 0.55 |
| 4h PAA | 25.35 | 1.08 |  | 24.00 | 0.1 |  | 26.43 | 0.4 |  | 26.40 | 1.02 |  | 25.93 | 0.39 |  | 23.33 | 0.26 |
| 6h | 20.05 | 0.54 |  | 21.28 | 0.2 |  | 21.40 | 0.5 |  | 20.35 | 0.41 |  | 23.55 | 0.18 |  | 22.88 | 0.26 |
| 6h PAA | 20.93 | 0.70 |  | 23.05 | 0.3 |  | 24.48 | 0.6 |  | 24.48 | 0.63 |  | 25.45 | 0.54 |  | 23.15 | 0.28 |
|  |  |  |  |  |  |  |  |  |  |  |  |  |  |  |  |  |  |
|  | ***ul7*** | |  | ***ul6*** | |  | ***ul5*** | |  | ***ul4*** | |  | ***ul3.5*** | |  | ***ul3*** | |
|  | **Ct** | **SE** |  | **Ct** | **SE** |  | **Ct** | **SE** |  | **Ct** | **SE** |  | **Ct** | **SE** |  | **Ct** | **SE** |
| 1h | 0.00 | 0.00 |  | 28.53 | 0.12 |  | 34.83 | 0.54 |  | 27.40 | 0.57 |  | 28.95 | 0.03 |  | 27.93 | 0.21 |
| 2h | 0.00 | 0.00 |  | 28.10 | 0.11 |  | 34.60 | 0.34 |  | 25.73 | 0.37 |  | 28.77 | 0.12 |  | 27.30 | 0.48 |
| 4h | 27.73 | 0.2 |  | 24.65 | 0.49 |  | 32.18 | 0.33 |  | 22.98 | 0.56 |  | 27.93 | 0.24 |  | 24.55 | 0.34 |
| 4h PAA | 28.15 | 0.4 |  | 27.00 | 0.11 |  | 33.80 | 0.32 |  | 24.03 | 0.21 |  | 28.80 | 0.00 |  | 26.38 | 0.26 |
| 6h | 26.65 | 0.1 |  | 22.58 | 0.39 |  | 31.07 | 0.35 |  | 21.68 | 0.15 |  | 27.18 | 0.29 |  | 22.98 | 0.39 |
| 6h PAA | 27.70 | 0.2 |  | 25.65 | 0.43 |  | 33.35 | 0.41 |  | 23.75 | 0.92 |  | 28.45 | 0.10 |  | 25.68 | 0.45 |
|  |  |  |  |  |  |  |  |  |  |  |  |  |  |  |  |  |  |
|  | ***ul2*** | |  | ***ul1*** | |  | ***ep0*** | |  | ***llt1 ep0*** | |  | ***llt2 ie180*** | |  | ***ie180*** | |
|  | **Ct** | **SE** |  | **Ct** | **SE** |  | **Ct** | **SE** |  | **Ct** | **SE** |  | **Ct** | **SE** |  | **Ct** | **SE** |
| 1h | 27.03 | 0.52 |  | 27.17 | 1.05 |  | 25.60 | 0.68 |  | 28.93 | 0.17 |  | 28.00 | 0.02 |  | 24.93 | 0.43 |
| 2h | 26.90 | 1.03 |  | 25.43 | 0.36 |  | 23.77 | 1.26 |  | 29.08 | 0.35 |  | 29.30 | 0.01 |  | 25.20 | 0.68 |
| 4h | 24.28 | 1.35 |  | 22.05 | 0.33 |  | 21.28 | 0.75 |  | 27.23 | 0.76 |  | 28.13 | 0.16 |  | 25.28 | 1.48 |
| 4h PAA | 24.53 | 0.25 |  | 24.73 | 0.45 |  | 23.00 | 0.40 |  | 28.93 | 0.28 |  | 28.73 | 0.25 |  | 25.57 | 0.21 |
| 6h | 20.60 | 0.35 |  | 19.03 | 0.63 |  | 20.98 | 0.54 |  | 24.63 | 0.46 |  | 27.10 | 0.41 |  | 22.68 | 0.59 |
| 6h PAA | 25.17 | 1.15 |  | 23.58 | 0.74 |  | 22.03 | 0.27 |  | 28.55 | 0.12 |  | 27.97 | 0.10 |  | 24.75 | 0.31 |
|  |  |  |  |  |  |  |  |  |  |  |  |  |  |  |  |  |  |
|  | ***us1*** | |  | ***us3*** | |  | ***us4*** | |  | ***us6*** | |  | ***us7*** | |  | ***us8*** | |
|  | **Ct** | **SE** |  | **Ct** | **SE** |  | **Ct** | **SE** |  | **Ct** | **SE** |  | **Ct** | **SE** |  | **Ct** | **SE** |
| 1h | 28.40 | 0.01 |  | 27.75 | 0.60 |  | 26.73 | 0.47 |  | 28.20 | 0.18 |  | 27.47 | 0.60 |  | 26.00 | 0.61 |
| 2h | 28.17 | 0.08 |  | 26.90 | 0.52 |  | 24.43 | 0.15 |  | 27.75 | 0.35 |  | 26.88 | 0.30 |  | 24.33 | 0.34 |
| 4h | 26.90 | 0.64 |  | 24.88 | 1.25 |  | 21.70 | 1.13 |  | 26.15 | 0.35 |  | 23.10 | 0.72 |  | 21.78 | 0.70 |
| 4h PAA | 28.08 | 0.08 |  | 24.90 | 0.13 |  | 23.33 | 0.33 |  | 26.80 | 0.18 |  | 25.18 | 0.42 |  | 23.38 | 0.15 |
| 6h | 24.10 | 0.39 |  | 24.07 | 0.92 |  | 20.20 | 1.17 |  | 23.45 | 0.46 |  | 20.88 | 0.22 |  | 19.55 | 0.17 |
| 6h PAA | 27.85 | 0.04 |  | 25.30 | 0.94 |  | 22.18 | 0.26 |  | 26.08 | 0.82 |  | 23.55 | 0.37 |  | 22.53 | 0.51 |
|  |  |  |  |  |  |  |  |  |  |  |  |  |  |  |  |  |  |
|  | ***us9*** | |  | ***us2*** | |  |  |  |  |  |  |  |  |  |  |  |  |
|  | **Ct** | **SE** |  | **Ct** | **SE** |  |  |  |  |  |  |  |  |  |  |  |  |
| 1h | 27.30 | 0.17 |  | 28.30 | 0.19 |  |  |  |  |  |  |  |  |  |  |  |  |
| 2h | 26.48 | 0.77 |  | 27.03 | 0.22 |  |  |  |  |  |  |  |  |  |  |  |  |
| 4h | 23.38 | 0.55 |  | 25.33 | 0.41 |  |  |  |  |  |  |  |  |  |  |  |  |
| 4h PAA | 25.08 | 0.17 |  | 26.40 | 0.22 |  |  |  |  |  |  |  |  |  |  |  |  |
| 6h | 21.38 | 0.47 |  | 22.68 | 0.26 |  |  |  |  |  |  |  |  |  |  |  |  |
| 6h PAA | 23.85 | 0.65 |  | 25.85 | 0.53 |  |  |  |  |  |  |  |  |  |  |  |  |

|  | ***orf-1*** | |  | ***ul54*** | |  | ***ul53*** | |  | ***ul52*** | |  | ***ul51*** | |  | ***ul50*** | |
| --- | --- | --- | --- | --- | --- | --- | --- | --- | --- | --- | --- | --- | --- | --- | --- | --- | --- |
| **Time** | **E ^a^** | **SE ^b^** |  | **E** | **SE** |  | **E** | **SE** |  | **E** | **SE** |  | **E** | **SE** |  | **E** | **SE** |
| **1h** | 1,89 | 0,05 |  | 1,70 | 0,01 |  | - | - |  | 1,82 | 0,02 |  | 1,84 | 0,01 |  | 1,73 | 0,01 |
| **2h** | 1,88 | 0,02 |  | 1,74 | 0,03 |  | - | - |  | 1,74 | 0,00 |  | 1,82 | 0,02 |  | 1,72 | 0,00 |
| **4h** | 1,67 | 0,05 |  | 1,70 | 0,00 |  | 1,76 | 0,08 |  | 1,70 | 0,01 |  | 1,67 | 0,01 |  | 1,70 | 0,01 |
| **4h PAA** | 1,72 | 0,02 |  | 1,70 | 0,01 |  | 1,80 | 0,03 |  | 1,67 | 0,04 |  | 1,76 | 0,01 |  | 1,74 | 0,03 |
| **6h** | 1,68 | 0,01 |  | 1,70 | 0,00 |  | 1,75 | 0,01 |  | 1,69 | 0,01 |  | 1,64 | 0,01 |  | 1,72 | 0,01 |
| **6h PAA** | 1,74 | 0,06 |  | 1,70 | 0,01 |  | 1,76 | 0,05 |  | 1,69 | 0,00 |  | 1,74 | 0,02 |  | 1,72 | 0,00 |
|  |  |  |  |  |  |  |  |  |  |  |  |  |  |  |  |  |  |
|  | ***ul49.5*** | |  | ***ul49*** | |  | ***ul48*** | |  | ***ul47*** | |  | ***ul46*** | |  | ***ul27*** | |
|  | **E** | **SE** |  | **E** | **SE** |  | **E** | **SE** |  | **E** | **SE** |  | **E** | **SE** |  | **E** | **SE** |
| **1h** | 1.90 | 0,04 |  | 1,69 | 0,03 |  | 1,72 | 0,01 |  | 1,87 | 0,02 |  | 1,71 | 0,01 |  | 1,74 | 0,01 |
| **2h** | 1,71 | 0,02 |  | 1,61 | 0,02 |  | 1,68 | 0,01 |  | 1,74 | 0,06 |  | 1,72 | 0,02 |  | 1,75 | 0,04 |
| **4h** | 1.65 | 0,04 |  | 1,69 | 0,01 |  | 1,69 | 0,02 |  | 1,65 | 0,02 |  | 1,69 | 0,01 |  | 1,67 | 0,02 |
| **4h PAA** | 1,70 | 0,02 |  | 1,71 | 0,03 |  | 1,68 | 0,01 |  | 1,76 | 0,05 |  | 1,70 | 0,01 |  | 1,67 | 0,01 |
| **6h** | 1,57 | 0,01 |  | 1,69 | 0,03 |  | 1,70 | 0,01 |  | 1,60 | 0,03 |  | 1,69 | 0,01 |  | 1,69 | 0,01 |
| **6h PAA** | 1,65 | 0,01 |  | 1,71 | 0,04 |  | 1,70 | 0,01 |  | 1,70 | 0,02 |  | 1,68 | 0,02 |  | 1,67 | 0,04 |
|  |  |  |  |  |  |  |  |  |  |  |  |  |  |  |  |  |  |
|  | ***ul28*** | |  | ***ul29*** | |  | ***ul30*** | |  | ***ul31*** | |  | ***ul32*** | |  | ***ul33*** | |
|  | **E** | **SE** |  | **E** | **SE** |  | **E** | **SE** |  | **E** | **SE** |  | **E** | **SE** |  | **E** | **SE** |
| **1h** | 1,70 | 0,04 |  | 1,69 | 0,01 |  | 1,73 | 0,07 |  | 1,77 | 0,02 |  | 1,82 | 0,02 |  | 1,74 | 0,01 |
| **2h** | 1,67 | 0,02 |  | 1,72 | 0,01 |  | 1,71 | 0,04 |  | 1,70 | 0,01 |  | 1,72 | 0,00 |  | 1,71 | 0,01 |
| **4h** | 1,61 | 0,01 |  | 1,72 | 0,01 |  | 1,78 | 0,04 |  | 1,72 | 0,01 |  | 1,70 | 0,01 |  | 1,69 | 0,01 |
| **4h PAA** | 1,64 | 0,01 |  | 1,73 | 0,01 |  | 1,74 | 0,04 |  | 1,71 | 0,01 |  | 1,72 | 0,01 |  | 1,69 | 0,01 |
| **6h** | 1,63 | 0,02 |  | 1,74 | 0,01 |  | 1,73 | 0,04 |  | 1,70 | 0,01 |  | 1,69 | 0,01 |  | 1,73 | 0,01 |
| **6h PAA** | 1,64 | 0,02 |  | 1,74 | 0,01 |  | 1,76 | 0,01 |  | 1,71 | 0,01 |  | 1,68 | 0,01 |  | 1,69 | 0,01 |
|  |  |  |  |  |  |  |  |  |  |  |  |  |  |  |  |  |  |
|  | ***ul34*** | |  | ***ul35*** | |  | ***ul36*** | |  | ***ul37*** | |  | ***ul38*** | |  | ***ul39*** | |
|  | **E** | **SE** |  | **E** | **SE** |  | **E** | **SE** |  | **E** | **SE** |  | **E** | **SE** |  | **E** | **SE** |
| **1h** | 1,71 | 0,01 |  | 1,70 | 0,01 |  | 1,71 | 0,01 |  | 1,84 | 0,05 |  | 1,76 | 0,04 |  | 1,73 | 0,01 |
| **2h** | 1,66 | 0,02 |  | 1,70 | 0,01 |  | 1,71 | 0,01 |  | 1,71 | 0,00 |  | 1,80 | 0,07 |  | 1,70 | 0,01 |
| **4h** | 1,66 | 0,01 |  | 1,70 | 0,01 |  | 1,71 | 0,01 |  | 1,72 | 0,01 |  | 1,70 | 0,03 |  | 1,69 | 0,01 |
| **4h PAA** | 1,72 | 0,01 |  | 1,70 | 0,01 |  | 1,72 | 0,01 |  | 1,71 | 0,01 |  | 1,84 | 0,04 |  | 1,67 | 0,01 |
| **6h** | 1,66 | 0,02 |  | 1,69 | 0,01 |  | 1,71 | 0,01 |  | 1,70 | 0,01 |  | 1,63 | 0,01 |  | 1,69 | 0,01 |
| **6h PAA** | 1,69 | 0,01 |  | 1,69 | 0,01 |  | 1,71 | 0,01 |  | 1,71 | 0,01 |  | 1,79 | 0,04 |  | 1,70 | 0,01 |
|  |  |  |  |  |  |  |  |  |  |  |  |  |  |  |  |  |  |
|  | ***ul40*** | |  | ***ul41*** | |  | ***ul42*** | |  | ***ul43*** | |  | ***ul44*** | |  | ***ul26*** | |
|  | **E** | **SE** |  | **E** | **SE** |  | **E** | **SE** |  | **E** | **SE** |  | **E** | **SE** |  | **E** | **SE** |
| **1h** | 1,72 | 0,01 |  | 1,87 | 0,02 |  | 1,73 | 0,02 |  | 1,76 | 0,04 |  | 1,84 | 0,02 |  | 1,79 | 0,05 |
| **2h** | 1,71 | 0,01 |  | 1,75 | 0,01 |  | 1,68 | 0,01 |  | 1,69 | 0,03 |  | 1,86 | 0,02 |  | 1,77 | 0,03 |
| **4h** | 1,75 | 0,03 |  | 1,74 | 0,02 |  | 1,68 | 0,00 |  | 1,69 | 0,02 |  | 1,71 | 0,02 |  | 1,70 | 0,02 |
| **4h PAA** | 1,69 | 0,02 |  | 1,70 | 0,01 |  | 1,69 | 0,01 |  | 1,69 | 0,02 |  | 1,80 | 0,03 |  | 1,75 | 0,04 |
| **6h** | 1,73 | 0,01 |  | 1,72 | 0,01 |  | 1,68 | 0,00 |  | 1,70 | 0,01 |  | 1,63 | 0,04 |  | 1,70 | 0,02 |
| **6h PAA** | 1,71 | 0,03 |  | 1,73 | 0,03 |  | 1,68 | 0,01 |  | 1,74 | 0,04 |  | 1,72 | 0,02 |  | 1,68 | 0,01 |
|  |  |  |  |  |  |  |  |  |  |  |  |  |  |  |  |  |  |
|  | ***ul25*** | |  | ***ul24*** | |  | ***ul23*** | |  | ***ul22*** | |  | ***ul21*** | |  | ***ul20*** | |
|  | **E** | **SE** |  | **E** | **SE** |  | **E** | **SE** |  | **E** | **SE** |  | **E** | **SE** |  | **E** | **SE** |
| **1h** | 1,89 | 0,03 |  | 1,79 | 0,01 |  | 1,72 | 0,02 |  | 1,72 | 0,00 |  | 1,72 | 0,01 |  | 1,75 | 0,03 |
| **2h** | 1,80 | 0,01 |  | 1,78 | 0,01 |  | 1,69 | 0,01 |  | 1,69 | 0,01 |  | 1,72 | 0,00 |  | 1,72 | 0,01 |
| **4h** | 1,71 | 0,01 |  | 1,73 | 0,01 |  | 1,69 | 0,01 |  | 1,68 | 0,01 |  | 1,72 | 0,01 |  | 1,66 | 0,01 |
| **4h PAA** | 1,71 | 0,01 |  | 1,73 | 0,02 |  | 1,69 | 0,01 |  | 1,68 | 0,01 |  | 1,73 | 0,01 |  | 1,65 | 0,02 |
| **6h** | 1,70 | 0,02 |  | 1,74 | 0,01 |  | 1,73 | 0,04 |  | 1,65 | 0,01 |  | 1,73 | 0,01 |  | 1,65 | 0,02 |
| **6h PAA** | 1,72 | 0,01 |  | 1,75 | 0,01 |  | 1,69 | 0,01 |  | 1,66 | 0,02 |  | 1,69 | 0,01 |  | 1,65 | 0,01 |

|  | ***ul19*** | |  | ***ul18*** | |  | ***ul17*** | |  | ***ul16*** | |  | ***ul15*** | |  | ***ul14*** | |
| --- | --- | --- | --- | --- | --- | --- | --- | --- | --- | --- | --- | --- | --- | --- | --- | --- | --- |
|  | **E** | **SE** |  | **E** | **SE** |  | **E** | **SE** |  | **E** | **SE** |  | **E** | **SE** |  | **E** | **SE** |
| 1h | 1,74 | 0,02 |  | 1,79 | 0,03 |  | 1,90 | 0,01 |  | - | - |  | 1,83 | 0,02 |  | 1,72 | 0,02 |
| 2h | 1,72 | 0,01 |  | 1,70 | 0,03 |  | 1,89 | 0,01 |  | - | - |  | 1,74 | 0,01 |  | 1,71 | 0,01 |
| 4h | 1,69 | 0,01 |  | 1,68 | 0,03 |  | 1,72 | 0,02 |  | 1,78 | 0,85 |  | 1,72 | 0,01 |  | 1,72 | 0,01 |
| 4h PAA | 1,69 | 0,01 |  | 1,71 | 0,01 |  | 1,80 | 0,01 |  | 1,50 | 0,00 |  | 1,70 | 0,01 |  | 1,70 | 0,01 |
| 6h | 1,71 | 0,01 |  | 1,71 | 0,01 |  | 1,70 | 0,01 |  | 1,73 | 0,07 |  | 1,70 | 0,00 |  | 1,73 | 0,01 |
| 6h PAA | 1,66 | 0,01 |  | 1,71 | 0,01 |  | 1,72 | 0,02 |  | - | - |  | 1,70 | 0,01 |  | 1,72 | 0,01 |
|  |  |  |  |  |  |  |  |  |  |  |  |  |  |  |  |  |  |
|  | ***ul13*** | |  | ***ul12*** | |  | ***ul11*** | |  | ***ul10*** | |  | ***ul9*** | |  | ***ul8*** | |
|  | **E** | **SE** |  | **E** | **SE** |  | **E** | **SE** |  | **E** | **SE** |  | **E** | **SE** |  | **E** | **SE** |
| 1h | 1,69 | 0,01 |  | 1,74 | 0,01 |  | 1,65 | 0,06 |  | 1,70 | 0,05 |  | 1,74 | 0,03 |  | 1,76 | 0,01 |
| 2h | 1,71 | 0,01 |  | 1,72 | 0,01 |  | 1,59 | 0,03 |  | 1,72 | 0,07 |  | 1,69 | 0,00 |  | 1,75 | 0,01 |
| 4h | 1,69 | 0,00 |  | 1,70 | 0,01 |  | 1,62 | 0,02 |  | 1,69 | 0,02 |  | 1,69 | 0,01 |  | 1,70 | 0,00 |
| 4h PAA | 1,72 | 0,02 |  | 1,69 | 0,01 |  | 1,63 | 0,01 |  | 1,72 | 0,01 |  | 1,69 | 0,01 |  | 1,72 | 0,01 |
| 6h | 1,69 | 0,02 |  | 1,72 | 0,01 |  | 1,66 | 0,03 |  | 1,66 | 0,02 |  | 1,70 | 0,01 |  | 1,70 | 0,01 |
| 6h PAA | 1,68 | 0,02 |  | 1,71 | 0,01 |  | 1,56 | 0,01 |  | 1,69 | 0,01 |  | 1,67 | 0,01 |  | 1,70 | 0,02 |
|  |  |  |  |  |  |  |  |  |  |  |  |  |  |  |  |  |  |
|  | ***ul7*** | |  | ***ul6*** | |  | ***ul5*** | |  | ***ul4*** | |  | ***ul3.5*** | |  | ***ul3*** | |
|  | **E** | **SE** |  | **E** | **SE** |  | **E** | **SE** |  | **E** | **SE** |  | **E** | **SE** |  | **E** | **SE** |
| 1h | - | - |  | 1,85 | 0,03 |  | 1,69 | 0,01 |  | 1,76 | 0,03 |  | 1,92 | 0,03 |  | 1,79 | 0,02 |
| 2h | - | - |  | 1,77 | 0,04 |  | 1,65 | 0,04 |  | 1,71 | 0,00 |  | 1,89 | 0,02 |  | 1,74 | 0,02 |
| 4h | 1,73 | 0,02 |  | 1,73 | 0,01 |  | 1,60 | 0,00 |  | 1,72 |  |  | 1,80 | 0,03 |  | 1,71 | 0,01 |
| 4h PAA | 1,89 | 0.07 |  | 1,74 | 0,01 |  | 1,60 | 0,01 |  | 1,73 | 0,00 |  | 1,95 | 0,01 |  | 1,71 | 0,01 |
| 6h | 1,69 | 0,01 |  | 1,73 | 0,01 |  | 1,58 | 0,02 |  | 1,73 | 0,01 |  | 1,73 | 0,01 |  | 1,69 | 0,00 |
| 6h PAA | 1,71 | 0,01 |  | 1,77 | 0,04 |  | 1,63 | 0,02 |  | 1,73 | 0,02 |  | 1,66 | 0,04 |  | 1,71 | 0,03 |
|  |  |  |  |  |  |  |  |  |  |  |  |  |  |  |  |  |  |
|  | ***ul2*** | |  | ***ul1*** | |  | ***ep0*** | |  | ***llt1 ep0*** | |  | ***llt2 ie180*** | |  | ***ie180*** | |
|  | **E** | **SE** |  | **E** | **SE** |  | **E** | **SE** |  | **E** | **SE** |  | **E** | **SE** |  | **E** | **SE** |
| 1h | 1,69 | 0,01 |  | 1,66 | 0,02 |  | 1,72 | 0,00 |  | 1,82 | 0,08 |  | 1,55 | 0,01 |  | 1,66 | 0,03 |
| 2h | 1,68 | 0,03 |  | 1,68 | 0,02 |  | 1,74 | 0,02 |  | 1,67 | 0,08 |  | 1,51 | 0,01 |  | 1,70 | 0,01 |
| 4h | 1,67 | 0,03 |  | 1,68 | 0,05 |  | 1,73 | 0,01 |  | 1,78 | 0,03 |  | 1,83 | 0,06 |  | 1,65 | 0,03 |
| 4h PAA | 1,66 | 0,01 |  | 1,69 | 0,06 |  | 1,73 | 0,01 |  | 1,79 | 0,08 |  | 1,55 | 0,01 |  | 1,70 | 0,01 |
| 6h | 1,67 | 0,02 |  | 1.63 | 0,05 |  | 1,75 | 0,01 |  | 1,72 | 0,03 |  | 1,71 | 0,04 |  | 1,66 | 0,03 |
| 6h PAA | 1,66 | 0,04 |  | 1,72 | 0,02 |  | 1,75 | 0,01 |  | 1,91 | 0,03 |  | 1,63 | 0,06 |  | 1,69 | 0,01 |
|  |  |  |  |  |  |  |  |  |  |  |  |  |  |  |  |  |  |
|  | ***us1*** | |  | ***us3*** | |  | ***us4*** | |  | ***us6*** | |  | ***us7*** | |  | ***us8*** | |
|  | **E** | **SE** |  | **E** | **SE** |  | **E** | **SE** |  | **E** | **SE** |  | **E** | **SE** |  | **E** | **SE** |
| 1h | 1,79 | 0,00 |  | 1,77 | 0,07 |  | 1,67 | 0,02 |  | 1,79 | 0,03 |  | 1,77 | 0,04 |  | 1,73 | 0,01 |
| 2h | 1,63 | 0,01 |  | 1,70 | 0,02 |  | 1,70 | 0,02 |  | 1,77 | 0,04 |  | 1,70 | 0,02 |  | 1,71 | 0,01 |
| 4h | 1,65 | 0,02 |  | 1,72 | 0,04 |  | 1,67 | 0,01 |  | 1,69 | 0,01 |  | 1,69 | 0,00 |  | 1,69 | 0,01 |
| 4h PAA | 1,67 | 0,01 |  | 1,70 | 0,00 |  | 1,62 | 0,01 |  | 1,68 | 0,01 |  | 1,67 | 0,03 |  | 1,71 | 0,00 |
| 6h | 1,61 | 0,00 |  | 1,69 | 0,01 |  | 1,65 | 0,04 |  | 1,67 | 0,03 |  | 1,68 | 0,01 |  | 1,71 | 0,01 |
| 6h PAA | 1,67 | 0,00 |  | 1,71 | 0,02 |  | 1,66 | 0,01 |  | 1,71 | 0,02 |  | 1,69 | 0,01 |  | 1,69 | 0,01 |
|  |  |  |  |  |  |  |  |  |  |  |  |  |  |  |  |  |  |
|  | ***us9*** | |  | ***us2*** | |  |  |  |  |  |  |  |  |  |  |  |  |
|  | **E** | **SE** |  | **E** | **SE** |  |  |  |  |  |  |  |  |  |  |  |  |
| 1h | 1,73 | 0,01 |  | 1,85 | 0,04 |  |  |  |  |  |  |  |  |  |  |  |  |
| 2h | 1,74 | 0,03 |  | 1,71 | 0,01 |  |  |  |  |  |  |  |  |  |  |  |  |
| 4h | 1,70 | 0,00 |  | 1,69 | 0,01 |  |  |  |  |  |  |  |  |  |  |  |  |
| 4h PAA | 1,71 | 0,01 |  | 1,71 | 0,01 |  |  |  |  |  |  |  |  |  |  |  |  |
| 6h | 1,69 | 0,01 |  | 1,68 | 0,01 |  |  |  |  |  |  |  |  |  |  |  |  |
| 6h PAA | 1,71 | 0,01 |  | 1,73 | 0,05 |  |  |  |  |  |  |  |  |  |  |  |  |
